# Advances in Electrospun Poly(ε-caprolactone)-Based Nanofibrous Scaffolds for Tissue Engineering

**DOI:** 10.3390/polym16202853

**Published:** 2024-10-10

**Authors:** Karla N. Robles, Fatima tuz Zahra, Richard Mu, Todd Giorgio

**Affiliations:** 1 TIGER Institute, Tennessee State University, Nashville, TN 37209, USA; zfatima56@gmail.com (F.t.Z.); todd.d.giorgio@vanderbilt.edu (T.G.); 2 Department of Biomedical Engineering, Vanderbilt University, Nashville, TN 37235, USA

**Keywords:** tissue engineering, scaffold fabrication, poly(ε-caprolactone) (PCL), electrospinning, biocompatibility, composite scaffolds, scaffold wettability, nanofibers, biomaterials

## Abstract

Tissue engineering has great potential for the restoration of damaged tissue due to injury or disease. During tissue development, scaffolds provide structural support for cell growth. To grow healthy tissue, the principal components of such scaffolds must be biocompatible and nontoxic. Poly(ε-caprolactone) (PCL) is a biopolymer that has been used as a key component of composite scaffolds for tissue engineering applications due to its mechanical strength and biodegradability. However, PCL alone can have low cell adherence and wettability. Blends of biomaterials can be incorporated to achieve synergistic scaffold properties for tissue engineering. Electrospun PCL-based scaffolds consist of single or blended-composition nanofibers and nanofibers with multi-layered internal architectures (i.e., core-shell nanofibers or multi-layered nanofibers). Nanofiber diameter, composition, and mechanical properties, biocompatibility, and drug-loading capacity are among the tunable properties of electrospun PCL-based scaffolds. Scaffold properties including wettability, mechanical strength, and biocompatibility have been further enhanced with scaffold layering, surface modification, and coating techniques. In this article, we review nanofibrous electrospun PCL-based scaffold fabrication and the applications of PCL-based scaffolds in tissue engineering as reported in the recent literature.

## 1. Introduction

Tissue engineering has wide biomedical applications (skin, vascular, bone tissue engineering, etc.) to restore or replace damaged tissue, fabricate artificial organs, and to create 3D disease models. Typically, tissue engineering employs cells seeded on scaffolds to derive tissue. Tissues are generally considered soft if their elastic moduli are within 1 kPa to 1 MPa. Brain, pancreas, kidney, muscle, skin, and nerve tissues are all considered soft tissues. Cartilage, ligaments, and bone are considered stiff or hard tissues [1]. Tissues vary in mechanical properties and behave anisotropically [1]. Tissues consist of cells, biological factors, and the extracellular matrix (ECM) existing interdependently. The ECM supports biochemical and mechanotransductive signals which impact cellular behavior (i.e., migration, differentiation, cell–cell signaling, etc.). The cells, in turn, deposit and remodel the ECM. Imbalances in cell-to-cell signaling, ECM properties, or intercommunication can result in the development of disease. ECM fibrils and fibers are made up of collagen, fibrin, fibronectin, laminin, fibrillin, and elastin. Other constituents of the ECM include polysaccharides such as glycosaminoglycans, which support its hydration and structural integrity.

ECM-mimicking scaffolds for tissue engineering are developed so specific tissue-related cells or stem cells are seeded, and the cells may infiltrate, remodel, and degrade the scaffold while depositing native ECM. The fabrication of such scaffolds should recapitulate native tissue biomechanics. Decellularized ECM (dECM) is biocompatible and can form 3D hydrogels; however, it lacks mechanical strength. Decellularized ECM-based composite scaffolds have been fabricated with synthetic polymers, natural polysaccharides, and natural proteins for tissue engineering [2]. Biopolymers such as collagen, gelatin, silk fibroin, and synthetic polymers such as PCL, PU, PLA, PEG, and PLGA are widely used biomaterials in tissue engineering scaffolds. Biopolymers such as gelatin or collagen have a globular structure that makes fiber fabrication challenging. Herbal extracts are commonly used materials in nanofibrous composites due to their antibacterial, antifungal, anti-inflammatory, antioxidant, and anticancer properties for wound care and skin tissue engineering [3]. Plant extracts have also been incorporated in tissue engineering scaffolds to attack tumor tissue and promote healthy tissue growth in breast cancer models [4].

**Figure 1:**
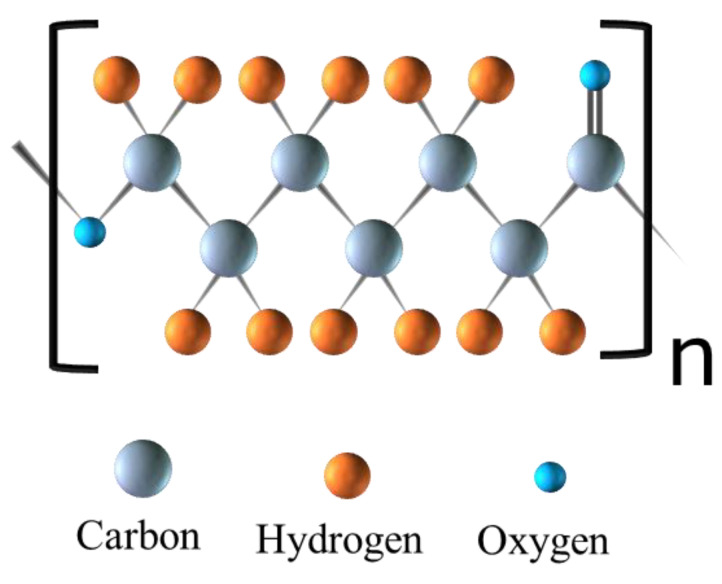
Chemical structure of poly(ε-caprolactone).

PCL is a hydrophobic synthetic polymer (Figure 1) with high mechanical strength that has been used as the basis for tissue engineering scaffolds [5,6,7]. PCL is a good candidate for tissue engineering due to its gradual degradation rate and mechanical strength. In vitro and in vivo studies of PCL for wound dressings have been performed to determine its degradation profile. PCL degrades more slowly in aqueous media (phosphate-buffered saline (PBS)) compared to enzymatic media in vitro. In vivo studies revealed the slowest degradation profile with some mild inflammatory markers. While the bioactivity of PCL alone is limited, PCL degrades in 90 days in enzymatic media in vitro. In in vivo studies, biointegration was demonstrated without complete degradation [8]. The mechanical strength at the nanoscale of most engineered tissues, such as bone and skin tissue, is in the range of 1.28–1.97 GPa, 4.5 MPa (epidermis), and 0.1 MPa (dermis), respectively [9]. Bulk PCL exhibits a tensile strength ranging from approximately 25 to 43 MPa and an elastic modulus between 330 and 360 MPa. In contrast, porous and fibrous PCL scaffolds demonstrate reduced tensile strength and elastic modulus, attributable to their porous architecture. Thus, while the mechanical strength and bioactivity of porous PCL scaffolds is suitable for skin tissue engineering, the incorporation of various ceramic materials into PCL blends enhances the mechanical properties of PCL scaffolds, thereby also making them suitable for applications in bone tissue engineering [10].

PCL properties, such as its degradation profile and mechanical strength, are adjustable by blending it with biomaterials, through surface-modification, or incorporating PCL-based scaffolds within hydrogels [11]. Various natural and synthetic materials have been blended with PCL to enhance the bioactivity of the composites, such as microcrystalline cellulose, gelatin, collagen, starch, polyvinyl pyrrolidone (PVP), polyvinyl alcohol (PVA), and poly(lactic-co-glycolic acid) (PLGA) [12,13,14,15,16,17]. Furthermore, aligned and randomly oriented electrospun PCL fibers on multiple scales have been shown to support cell proliferation and organization. Nano-size aligned fibers significantly enhance endothelial cell proliferation, and micro-size and aligned fibers have a guiding effect on cell migration and orientation [18]. At various concentrations of PCL, fibers appropriate for mimicking the morphologies of fibers from the spinal cord and pituitary gland have been produced. For these fibers, the tensile modulus increased with PCL concentration, but the wettability of the scaffold did not. Cell infiltration, however, was possible only in some scaffolds [19]. The mechanical strength of PCL is counterbalanced by its hydrophobicity and weak cell adherence. Alternatively, despite their excellent biocompatibility, natural polymers can result in heterogeneous scaffolds with poor mechanical properties and quick degradation rates. Like natural polymers, hydrophilic polymers risk degrading in aqueous cell culture media before cells have established a native extracellular matrix. However, the ability of natural and hydrophilic polymers to support cell viability should not be understated. In fact, many electrospun scaffolds for tissue engineering are composite scaffolds with emergent synergistic properties [20,21].

**Figure 2:**
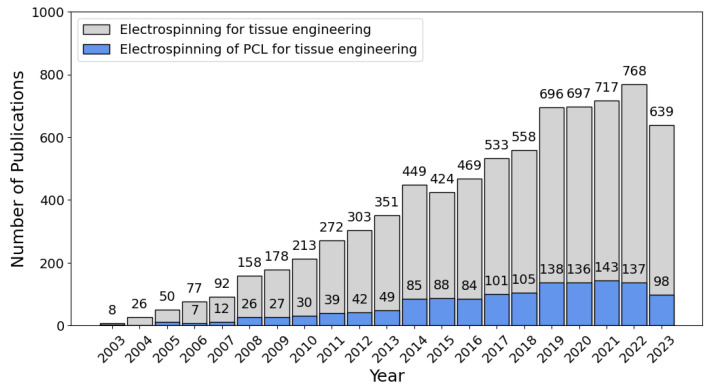
Publications from the last two decades listed on Web of Science containing keywords “electrospinning” and “tissue engineering” and “PCL”, “electrospinning”, and “tissue engineering”.

Composite scaffold fabrication to harness the synergy of mechanical strength from synthetic polymers with the biocompatible properties of biopolymers has been widely adopted due to its effectiveness [22,23]. Materials frequently used in electrospun nanofibrous scaffolds for skin tissue engineering include chitosan, silk fibroin, gelatin, PLA, and PCL [8,24,25,26]. In the last two decades, the number of publications on Web of Science regarding PCL, electrospinning, and tissue engineering has increased, as shown in Figure 2.

Electrospun nanofiber-enhanced hydrogel composite scaffolds [27], nanofibrous electrospun scaffolds for bone tissue engineering [28], and electrospun nanofibrous materials for hard and soft tissue engineering have been reviewed [29]. Recently, PCL in general health-care applications has also been reviewed [30]. However, no recent reviews with a specific focus on PCL-based scaffolds for tissue engineering have been reported, to the best of our knowledge. The review paper elucidates the importance of PCL nanofibers within the domain of tissue engineering, drawing upon recent studies in this area. It aims to serve as a comprehensive resource for readers seeking a deeper understanding of fundamentals pertinent to this field. We have reviewed electrospinning of PCL-based scaffold fabrication and enhanced functionalization techniques, as well as applications in tissue engineering, for recent publications from 2023 to present.

## 2. Background on Electrospinning

Electrospinning is a widely used nanofiber fabrication technique for the development of tissue engineering scaffolds [7,8]. Scaffolds are fabricated by collecting nanofibers continuously deposited onto a collector [22]. Nanofibrous scaffolds have been fabricated and characterized according to their nanofiber composition, wettability, mechanical properties, biological compatibility, biodegradability, and porosity [20,21]. Each of these characteristics is tunable, optimizable, and often interdependent. The electrospinning technique is used for tunable fabrication of advanced fibrous nanostructures and polymeric scaffolds [19,27,29]. Because it is highly tunable, electrospinning often requires thorough systematic optimization to achieve the desired scaffold properties [22,28].

**Figure 3:**
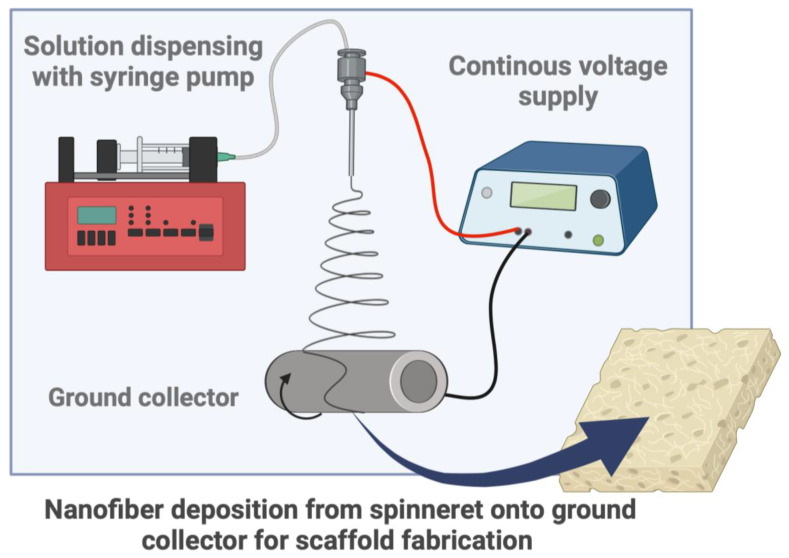
Schematic of fabrication of a nanofibrous scaffold with the electrospinning technique. Electrospinning set-up with syringe pump, steel emitter, voltage supply, and ground collector.

Electrospinning consists of a polymer solution dispensed at a constant flow rate with a syringe pump into a steel emitter or needle. At the same time, the needle receives an applied voltage from a voltage supply. Microjets emerge from the droplet as the voltage supply overcomes the surface tension of the solution. Fibers from the microjets are deposited on a ground collector as the solvent evaporates. Nanofibrous scaffolds are formed through continuous fiber deposition onto the collector (Figure 3).

Solvent and material properties should be considered to achieve a desired solution for electrospinning. Solvent properties include conductivity, volatility, and viscosity. Polymer properties include solvent solubility, polymer concentration, and polymer molecular weight. In coaxial electrospinning, immiscible or mostly immiscible solvents should be chosen to prevent solution intermixing during jet formation to ensure multi-layer structure fabrication.

Flow rate, needle gauge, distance to collector, applied voltage, and duration of electrospinning are parameters that must be optimized toward suitable nanofiber and scaffold properties. Fiber diameter can be reduced with a low flow rate, high needle gauge, increased distance to collector, and high applied voltage. In electrospinning, the collector type is also a parameter that may be adjusted from a flat conducting surface to a rotating cylinder.

Modifications to the electrospinning set-up have been reported to modify scaffold properties. A dual syringe with opposite applied voltages electrospun into a concentrator was used to fabricate twisted nanofiber-based yarns [25]. Multiple syringes on opposite sides of a cylindrical collector have also been used to fabricate multi-layer scaffolds of various fiber compositions [31]. A N_2_ flush from a mesh covering the collector during the electrospinning has been used to produce fine nanofiber-layered and organized porous scaffolds with high surface-to-volume ratio without a sacrificial polymer or agent. Histological analysis showed cell infiltration into inner fibrous layers [32].

Multi-scale computational models can assist in simulating and modeling scaffold properties. Electrospinning is highly tunable, and consequently, it has a large nonlinear parameter space. The optimization of electrospinning parameters with machine learning to predict nanofiber properties has been investigated [33]. Finite-element analysis has also been used to simulate the emergent mechanical behavior of scaffolds, including viscoelasticity and hyper-elasticity [34].

### 2.1. Tunable Electrospun PCL-Based Nanofiber Properties

The fiber diameter, fiber-to-fiber distance, fiber orientation, and composition in electrospun PCL-based scaffolds are tunable properties.

Kimura et al. reported a decrease in median fiber diameters from 429.7 nm after 1 h of electrospinning to 286.2 nm after 3 h [8]. Simultaneous electrospinning of sacrificial nanofibers with PCL-based nanofibers can increase the fiber-to-fiber distance [35]. With rotating cylinder collectors, the diameter and rotation speed influence scaffold properties. Randomly oriented electrospun PCL fibers were achieved using rotation speeds less than 100 RPM and controlled lateral movement [35]. Additionally, bilayer scaffolds with a bottom layer of aligned fibers and a top layer of randomly oriented fibers have been fabricated using a collector with a unique geometry [36].

**Figure 4:**
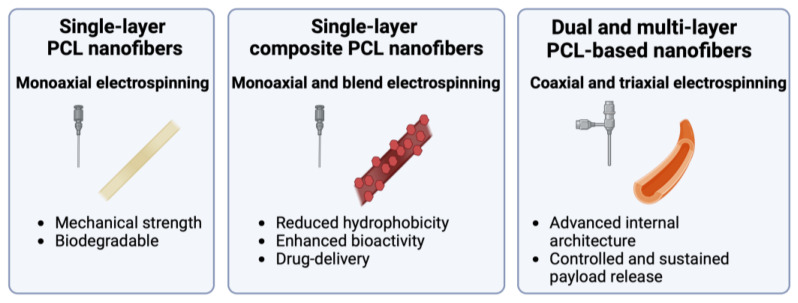
Types of PCL-based nanofibers fabricated with electrospinning.

Nanofiber properties can be further modified by introducing additional materials, therapeutic or otherwise, to the electrospinning solution. Composite PCL nanofibers are commonly fabricated by incorporating biological/natural products [8], additional synthetic polymers [21], bioactive nanoparticles [37], and/or drug loads [38] with PCL through monoaxial, blend, or co-electrospinning [21]. Variations such as coaxial and triaxial electrospinning involve separate immiscible solutions flowing through concentric needles to fabricate core-shell or multilayered fibers [21] (see Figure 4). Coaxial electrospinning is used to fabricate core-shell nanofibers, where PCL can serve distinct roles, including as a structural component in the core or as a sustained-delivery drug carrier in the shell. A summary of biomaterials frequently blend-electrospun with PCL is provided in Table 1.

In summary, the properties of electrospun PCL-based nanofibers include nanofiber diameter, nanofiber orientation, fiber-to-fiber distance, material composition, and drug release. These influence the properties of the emergent scaffold.

### 2.2. Electrospun PCL-Based Nanofibrous Scaffolds

**Figure 5:**
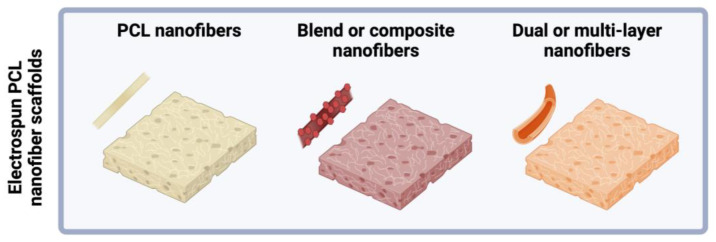
Types of emergent scaffolds fabricated from electrospun pristine PCL nanofibers, blend or composite nanofibers, and dual or multi-layer nanofibers.

Scaffolds can be fabricated with single-layer PCL nanofibers, composite PCL nanofibers, or core-shell and multilayered nanofibers (Figure 5). Characteristics of electrospun PCL scaffolds, including porosity, wettability, mechanical strength, and biocompatibility, can be optimized through the electrospinning process parameters and with the incorporation of additional materials.

The porosity of scaffolds impacts the transport and diffusion of nutrients, oxygen, and waste during tissue development. Scaffold porosity also affects cell migration and proliferation. The porosity of fibrous scaffolds can be controlled and tuned through the modulation of fiber diameter and the density of fiber packing [55,56].

Synthetic and composite polymeric fibers can be fabricated on multi-scale ranges from nano (1–1000 nm), micro (1–1000 µm), and macro (>100 µm) [4]. Liu et al. fabricated PCL nanofibers by spraying onto negatively charged (−1 kV) rotating cylindrical rods at various RPMs (200, 1100, 2000) and used laser metrology techniques to investigate porosity and pore size gradients of the nanofiber deposition. Rod-to-rod variability in porosity (%) was greatest at the lowest RPM. Pore size was reduced by increasing rotation speed or increasing negative voltage cylindrical bias and varied with deposition location on the cylinder. A −1 kV bias, compared to 0 kV, at 200 RPM reduced pore size from ~54 to ~37 µm. A bias of −5 kV resulted in a further reduced pore size of ~19 µm. Pore size was reduced from ~37 µm to ~23 µm at −1 kV cylindrical bias when increasing RPM from 200 to 2000 [27].

Physical properties of scaffolds such as wettability and mechanical properties can also be optimized by electrospun nanofiber composition. For example, a study investigated pristine PCL fibers, coaxial PVA shell-PCL core fibers, and tri-layer PVA-PCL-PVA scaffolds to enhance wettability and cell affinity. The study assessed the mechanical properties, cell affinity, and degradation of the scaffolds. Coaxial fibers exhibited significant variability in fiber diameter, and the core-shell architecture was evident. Wettability measurements showed that PCL scaffolds had a contact angle of 119° ± 2°, the tri-layer scaffolds’ water contact angle was ~21° ± 3°, and for the core-shell fibers the water contact angle was 43° ± 2°. After 28 weeks, the core-shell fiber scaffold demonstrated the highest water uptake and weight loss. Stress–strain curves indicated that Young’s modulus and ultimate tensile strength increased with degradation time, while maximum strain at failure decreased. At 48 h of incubation, all scaffolds had similar rates of cell viability for HEK-293 cells, as determined by the MTT assay. SEM images after 7 days of culture revealed varying cell morphologies, with cells spreading in the core-shell fiber scaffold. Fluorescent microscope images of DAPI (nucleus) stain showed the greatest cell growth and adherence on the core-shell scaffold [21].

In summary, electrospun PCL-based scaffolds can be engineered to have tunable porosity, wettability, mechanical properties, and biocompatibility.

### 2.3. Biomaterials Blend-Electrospun with PCL for Enhanced Scaffold Properties

**Figure 6:**
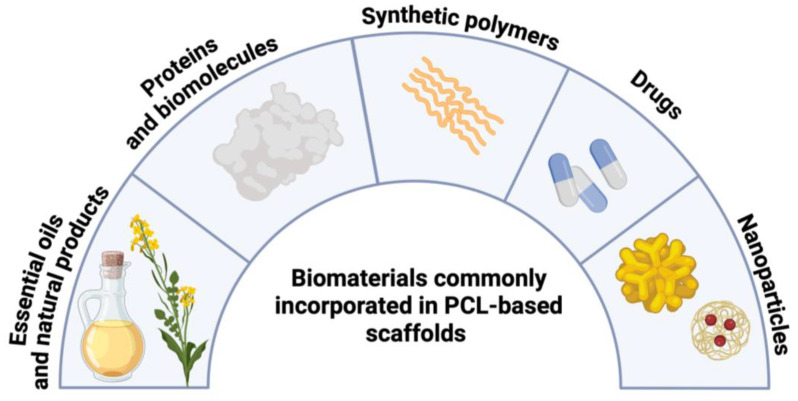
Overview of biomaterials commonly blend-electrospun with PCL to fabricate composite PCL-based scaffolds.

**Figure 7:**
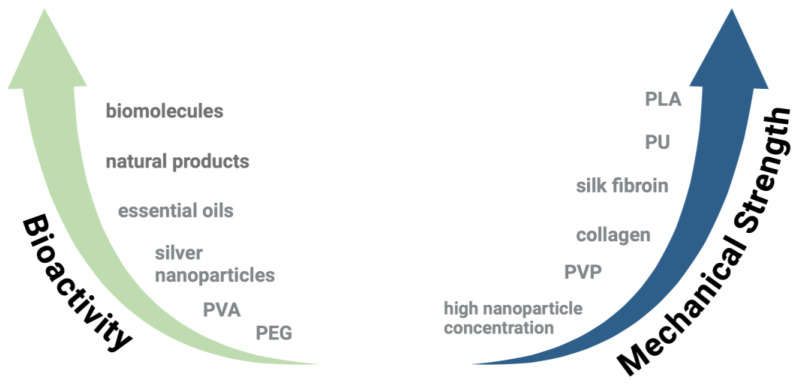
Overview of biomaterials commonly blend-electrospun with PCL and their relative influence on scaffold bioactivity and mechanical strength.

A wide array of biomaterials has been blend-electrospun with PCL to enhance scaffold properties. Figure 6 shows an overview of the biomaterials such as essential oils and natural products, proteins and biomolecules, synthetic polymers, drugs, and nanoparticles, and Figure 7 summarizes their influence on scaffold bioactivity and mechanical strength. The benefits and disadvantages of commonly used biopolymers and synthetic polymers are listed in Table 1.

#### 2.3.1. Gelatin

Gelatin is a protein that is a naturally derived, degraded form of collagen. It supports cell attachment, proliferation, and wound healing but has limitations, such as low spinnability, rapid degradation, and low mechanical strength. To address these issues, gelatin is often electrospun with PCL to improve scaffold properties such as porosity, surface wettability, mechanical strength, and biocompatibility [7,8]. It has been used with PCL-based scaffolds in formulations with borate glass [57], gold nanoparticles [58], titanium dioxide [59], chitosan [24], and surface modifications [60], as a coating [61], within a hydrogel scaffold [62], and in multi-layer scaffolds [63]. When used as a coating or filler, it has been used for growth-factor delivery [64].

Optimal conditions for the fabrication of gelatin-PCL sponges have been investigated using varying PCL concentrations in a 1:1 acetic acid to formic acid solution, with gelatin ratios of 60:40 and 80:20. The resulting gelatin-PCL sponges had a higher porosity (96% vs. 89%) and a lower water contact angle (37° compared to 95°), while supporting cell growth [7]. Gelatin-PCL scaffolds of varying thicknesses can be fabricated by adjusting the duration of electrospinning. SEM images suggest nanofiber size on the scaffold surface decreases with deposition times, consistent with other reports [8]. All gelatin-PCL scaffolds had water contact angles between 31° and 42°. Mechanical tests indicated increased strain at maximum load with longer deposition times except for the longest deposition of 3 h [8]. Fish gelatin, electrospun with PCL, produces nanofibrous (500–750 nm diameter) scaffolds. Increasing PCL concentration improved scaffold mass retention, and degradation was monitored over 14 days. Stress–strain curves suggest that mass ratio, scaffold environment (simulated body fluid or dry), and thermal crosslinking of fish gelatin with PCL affects the tensile mechanical properties of the scaffold [65].

In summary, gelatin is a frequently used material that can be employed in electrospun PCL-based scaffolds to achieve synergistic effects on tissue engineering. The studies are consistent in the reduction of water contact angle and support of cell attachment with the presence of gelatin in PCL-based scaffolds.

#### 2.3.2. Collagen

Collagen is a structural component of connective tissues. Unlike gelatin, collagen in its native form does not form a gel and hydrolyzed collagen is water soluble. It has been used both to blend-electrospin nanofibrous scaffolds and as a gel or coating agent [42,43,44]. There is a slight reduction in fiber diameter from PCL to collagen-PCL fibers [35].

#### 2.3.3. Silk Fibroin

Silk fibroin is a natural product from silkworm silk that is biodegradable and biocompatible, but its globular nature makes it challenging to electrospin. Silk fibroin increases biodegradation and biocompatibility in PCL-based scaffolds while also promoting mechanical strength [25,45,46].

#### 2.3.4. Other Natural Products

This section provides an overview the incorporation of natural products in PCL-based scaffolds, including propolis, chitosan, essential oils, and herbal extracts.

Propolis, a biological product used by bees in beehive construction, has favorable mechanical, structural, antibacterial, and antifungal properties. Uniform bead-free nanofibers with average diameter of 193 ± 31 nm have been achieved. The presence of propolis reduced the water contact angle and increased cell viability compared to plain PCL scaffolds [66]. When blend-electrospun with PCL and chitosan, propolis decreased fiber diameter and pore size and increased ultimate tensile strength and elongation at break. PCL, PCL-chitosan, and PCL-chitosan-propolis nanofibrous mats were blend-electrospun. Fiber diameter decreased and the number of pores decreased with propolis. Ultimate tensile strength, elongation at break, and water contact angle decreased with chitosan and increased with propolis. At the end of 48 h and 72 h, cell viability was greatest when cells were cultured on the propolis-loaded PCL chitosan mats [40]. Erdogmus et al. investigated the production of chitosan from the fungus *Rhizopus oryzae* for the fabrication of ultrathin chitosan-PCL nanofibers with average diameter of 100–120 nm [39]. Pectin, a fruit-derived polysaccharide with antimicrobial, biocompatible, and biodegradable properties, has also been blend-electrospun with PCL and chitosan [24,31].

Essential oils and herbal products have also been incorporated into PCL scaffolds. *Calophyllum inophyllum* oil, derived from the tropical family of trees, Calophyllaceae, is used in traditional herbal medicine to treat skin injuries. In blend-electrospinning, it increased fiber diameter and swelling capacity and decreased the water contact angle as low as 30° ± 5° [67]. Arbutin, found in arbutin leaves, is an antioxidant and hydrophilic. PCL-gelatin and PCL-gelatin arbutin scaffolds were fabricated by blend electrospinning and indicated good biocompatibility [68]. Genistin, from legumes, has anti-inflammatory, antibacterial, antimetastatic and estrogen-regulating properties. It was encapsulated in PCL microcapsules and electrospun into nanofibers and promoted proliferation and cell adhesion of L929 fibroblasts [63].

Platelet-rich plasma (PRP), egg white, and vitamin E are used in regenerative medicine due to their cell growth and repair properties. PRP, rich in growth factors, reduced nanofiber diameter, tensile strength, and water contact angle in PCL nanofibers. The scaffolds supported cell growth and showed non-toxicity in skin cells [69]. Egg white and vitamin E, despite increasing scaffold water contact angle, support cell adhesion and penetration. Egg whites attribute their viscosity to the protein albumin, which is also a rich nutrient supply for cell growth and repair. In one study, PCL, gelatin, and egg white were blend-electrospun. The presence of egg white increased average fiber diameter and increased water contact angle from 26° ± 7° to 116° ± 4°. The egg white-gelatin scaffolds had a positive effect on fibroblast growth measured on days 3 and 7 after seeding, perhaps attributed to albumin content. Adhesion and penetration of the scaffold of cells into the scaffold were evident in egg white-containing scaffolds [70]. In another study, a synthetic form of vitamin E, tocopheryl acetate, and silk fibroin were coaxially electrospun with PCL to fabricate antioxidant wound dressings. The nanofibers consisted of a PCL core and silk fibroin and tocopheryl acetate shell. Like with egg white, the presence of tocopheryl acetate reduced the wettability of the fibers, yet the tissue reconstruction was greatest in the PCL-silk fibroin-tocopheryl acetate group [46].

In short, propolis enhanced the mechanical properties of PCL-based scaffolds, including ultimate tensile strength and elongation at break. Natural products such as chitosan, essential oils and herbal products support cell growth, adhesion and repair.

#### 2.3.5. Synthetic Polymers

PVA has been coaxially electrospun with PCL to fabricate composite and multilayered nanofibers. The coaxially electrospun core-shell PCL-PVA fibers had increased cell affinity compared to PCL fiber scaffolds [71]. Cardenas et al. synthesized PCL and the block co-polymer PEO-b-PCL. H-NMR spectra showed peaks consistent with functional groups on both polymers. Of all the PCL synthesized, only the two PCL groups with the highest estimated molecular weight produced fibers. To the PCL solutions, 2.4% (*w*/*w*) of the block co-polymer PEO-PCL was added, and fibers with average diameter of 2–7 µm were produced. Water contact angle increased with PCL molecular weight and decreased with increasing PEO content. Young’s modulus increased and then decreased with increasing PEO content [72].

PLA and PCL were blend-electrospun with *Nigella stevia* extract to fabricate nanofibrous antibacterial scaffolds [33]. The PLA and PCL co-polymer has also been used in electrospun scaffold fabrication [38]. Oztemur et al. investigated the effect of PCL and PLA or PCL and PLLA blends on scaffold fabrication. The average fiber diameter ranged from 0.747 to 1.685 µm, with PLLA/PCL at 50:50 resulting in the lowest average fiber diameter and PLA resulting in the highest. Most formulations averaged a pore size in the range of 4–7 µm, except for the PLLA/PCL (20:80) blend, which averaged pore sizes of ~24 µm. The incorporation of PLA or PLLA both increased scaffold degradation rates, with the PLA50/PCL50 showing the greatest degradation (13.33%) at 5 months compared to PCL alone (3.70%). In cell viability studies (7 days), PLA20/PCL80 had the greatest fibroblast and human umbilical vein endothelial cell (HUVEC) viability, reported at days 3 and 7. Cell proliferation decreased with increasing PLA ratio, as high ratios of PLA harden the scaffold structure [54]. PLA-PCL co-polymer scaffolds either freestanding or incorporated on a 3D-printed framework, have also been electrospun for drug delivery. In in vitro studies, cell viability increased and then decreased on day 7 when exposed to varying concentrations of free drug. Ultimately, cells incubated on drug-loaded PLA-PCL-based scaffolds nearly doubled and tripled in cell viability, as measured with an MTT assay, compared to control and plain PLA-PCL-based scaffolds [38].

Electrospun PCL-PLGA blends have also been fabricated, and the water contact angle, mechanical strength, fiber diameter, and cell viability and pluripotency of iPSCs were characterized. Scaffolds composed of majority PLGA showed the greatest Young’s modulus. However, the water contact angle for all electrospun formulations was greater than 90 degrees. Of note, scaffolds fabricated with the same solutions with solvent casting displayed higher hydrophilicity and softness. Solvent-cast scaffolds showed higher cell viability at 5 days. Both fabrication techniques resulted in scaffolds that maintained iPSC pluripotency [66]. Other polymers used include poly(butylene succinate) (PBS) [20] and poly(glycerol sebacate) (PGS) [69].

Overall, various polymers have been blended and coaxial-electrospun to enhance the mechanical properties and biocompatibility PCL-based scaffolds. Coaxial electrospinning of PCL and PVA improved cell affinity compared to PCL alone. The inclusion of block co-polymer PEO-b-PCL in PCL solutions influences fiber diameter, water contact angle, and Young’s modulus. PLA, PLLA, and PLGA blends influence nanofiber diameter, pore size, degradation rates, and biocompatibility.

#### 2.3.6. Nanoparticles

Nanoparticles and nanowires are commonly used to enhance the properties of PCL-based scaffolds [44,53,73]. Silver nanoparticles, for example, have antibacterial properties that have been incorporated into PCL scaffolds [53,74]. In another study, *Moringa oleifera* leaf extracts were blend-electrospun with PCL and silver nanoparticles. In vitro analyses revealed zone inhibition of *S. aureus* and *E. coli* [75]. Silver-iron (Ag-Fe) nanoparticles have been synthesized from *Drucosia anethifolia* plant powder, silver nitrate, and iron(III) chloride hexahydrate and loaded onto PCL nanofibers. Ag-Fe nanoparticle-loaded scaffolds exhibited antibacterial and antifungal properties [37]. In PCL scaffolds loaded with zinc oxide (ZnO) nanowires, antimicrobial effects increased with ZnO concentration and exposure time. About 50% of *S. aureus* was sterilized with the ZnO-PCL scaffolds, demonstrating potential for antibacterial applications [76]. In another study, manganese oxide (MgO) nanoparticle-loaded PCL fibers were fabricated with blend-electrospinning. MgO particles served as an antibacterial agent [44].

Titanium oxide is a bio-inert and biocompatible ceramic that can enhance the mechanical properties of a scaffold. Titanium oxide nanoparticles were blend electrospun with PCL and gelatin to fabricate composite nanofibers. Nanofibers fabricated with titanium dioxide had lower average diameters and were more hydrophilic than nanofibers without titanium dioxide. The mechanical properties, including force, stress, elongation at break, and modulus of composite fibers, had a biphasic behavior that increased and then decreased with titanium oxide particle concentration [59].

Borate glass particles are bioactive and used in soft and hard tissue engineering. They were blend-electrospun at concentrations of 0%, 5%, and 10% with PCL and gelatin. Average fiber diameter increased with the mass fraction of borate glass particles. While PCL-gelatin scaffolds alone had a water contact angle of (45.0° ± 0.7°), which decreased with increased borate glass (30.0° ± 1.4°) [57].

Tantalum (Ta) and Whitlockite (WH) nanoparticles were blend-electrospun with PCL to fabricate a PCL-based scaffold to support neurovascular coupling. Tantalum nanoparticles have been shown to enhance osteoconduction, and WH releases Mg^2+^, which can increase nerve density. The incorporation of Ta/WH nanoparticles had no impact on scaffold surface wettability and decreased tensile properties [77].

PCL-carbon nanofiber scaffolds were fabricated by blend electrospinning PCL and carbon-nanofibers or by dip-coating into a carbon nanofiber solution. Cytotoxicity increased with carbon nanofiber concentration. PCL scaffolds that were surface-coated with carbon nanofibers showed lower cell proliferation compared to pristine PCL scaffolds alone. PCL scaffolds blend-electrospun with carbon nanofibers, however, had comparable cell proliferation rates to pristine PCL scaffolds [78].

In summary, nanoparticles and nanowires are frequently used to enhance scaffold mechanical properties and biocompatibility. Silver, silver-iron, and zinc oxide can be employed to introduce antibacterial properties to PCL-based scaffolds. With some nanoparticles, such as titanium dioxide and TaWH, mechanical strength is affected by increasing particle concentration.

## 3. Techniques for Enhancement of PCL-Based Scaffold Properties

**Figure 8:**
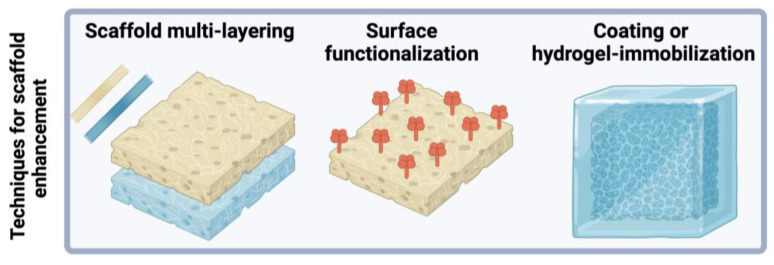
Overview of scaffold post-electrospinning processing techniques for enhanced functionality or biocompatibility.

To expand on the progress of electrospun PCL-based scaffolds fabricated with biomaterial blends, scaffold properties can be modified and optimized with multiple scaffold layers and chemical treatments post-electrospinning, such as surface functionalization, coating and hydrogel immobilization (see Figure 8). Table 2 summarizes the effects of scaffolds enhancement and post-electrospinning techniques on scaffold properties.

### 3.1. Multilayered Scaffold Fabrication

Multi-layered scaffolds can be readily employed by replacing the electrospinning solution or modifying electrospinning parameters. Dual- and tri-layer scaffolds have been fabricated to improve mechanical properties, cell viability, antibacterial properties, and oxygen delivery.

Silk fibroin has been blend-electrospun with PCL to fabricate dual-layer nanofibrous scaffolds. Separate layers were fabricated at PCL-SF ratios of 3:7 and 7:3 individually and as a stacked dual-layer scaffold. Silk fibroin-PCL fiber blends had improved tensile stress compared to pristine PCL scaffolds. The dual-layer scaffold exhibited the highest cell viability compared to silk fibroin or PCL nanofibers alone [45].

Two natural oils from the *Momordica charantia* and *Hypericum perforatum* plants were blend-electrospun with PCL. Separately, collagen and PCL were blend-electrospun. The PCL-oil and PCL-Col formulations produced fibers with average diameters of 921 ± 328 nm and 173 ± 54 nm, respectively. The PCL-oil and PCL-Col formulations had water contact angles of 97.8° and 11.7°. Then, the PCL-oil overlaid the PCL-collagen layer to create a dual-layer scaffold. On average, cell viability was lower for cells cultured on the scaffold than for untreated controls. The PCL-oil layer exhibited antibacterial properties [42].

A tri-layer scaffold was fabricated with the first layer of PCL and silver nanoparticles blend-electrospun. The second layer was also blend-electrospun and consisted of PCL and chitosan oligosaccharides spun onto the first layer. Then, the PCL bilayer was evenly coated with chitosan oligosaccharides and PVP. The tri-layer scaffold had lower mechanical strength when compared to PCL alone. Yet, the scaffold had improved wettability with a water contact angle of 34.77 compared to a PCL-Ag single-layer water contact angle of 116.67 [53]. In another study, tri-layer scaffolds were fabricated by electrospinning sequentially. First, alginate-dopamine and PVA were electrospun onto a rotating cylindrical collector. The resulting tubular scaffold was crosslinked. Then, the second layer, made of PCL and PVA, was electrospun onto the first layer. A third layer made of alginate-dopamine and PVA was electrospun onto the first two layers. Polydopamine nanoparticles were embedded in the first and third layers of the scaffold to improve cell adhesion through the introduction of catechol moieties [52]. In another study, a tri-layer PEO-PCL-PEO scaffold was fabricated by electrospinning. The PEO layer contained neem oil and *Hypericum perforatum* extracts [86]. Tri-layer oxygen-releasing scaffolds composed of polyurethane (PU) (layer 1), gelatin, PCL, and various peroxide concentrations (0%, 2.5%, 5%) (layer 2), and PU (layer 3) have also been fabricated [79].

In short, multi-layered scaffolds can improve mechanical properties, cell viability, and additional scaffold functionality, including antibacterial and oxygen-delivery properties. Dual-layer scaffolds with silk fibroin and PCL nanofibers had improved tensile stress and biocompatibility. However, tri-layer scaffolds incorporating additional materials, such as silver nanoparticles, chitosan oligosaccharides, and polydopamine, often had reduced mechanical strength compared to single-layer scaffolds.

### 3.2. Surface Modification or Functionalization

Surface modification techniques are employed to alter scaffold properties, such as porosity and surface wettability. In some studies, PCL-based scaffolds can better support tissue engineering if surface wettability is increased, as low surface wettability can diminish cell attachment, infiltration, and bioactivity. Surface modification of PCL-based scaffolds is carried out using wet chemical modification, surface graft polymerization, and plasma treatment.

PLLA-PCL nanofiber blends were fabricated, and the resultant scaffolds were treated with acetone to induce the formation of porous nanofiber surfaces. Acetone-treated PLLA-PCL scaffolds had lower water contact angles than non-treated groups. Young’s modulus and stress increased in acetone-treated PLLA-PCL scaffolds compared to non-treated groups, while strain decreased. Cell adhesion and proliferation were also improved in the acetone-treated groups [80]. Poly(l-lactide)/PCL electrospun blends were untreated, plasma-treated, or surfactant-treated and characterized for their hydrophilicity (i.e., water contact angle) and biocompatibility. Polymer fiber sponges were fabricated and treated with Tween 20, then optionally plasma-treated. Surfactant-treated sponges fully absorbed water and supported cell penetration into the scaffold. Plasma-treated scaffolds remained hydrophobic beneath their surface [81]. Sodium-alginate and PCL were emulsified, and nanofibers were electrospun. Then, the scaffolds were cross-linked and post-treated with calcium chloride to transform the sodium-alginate into calcium-alginate. This resulted in a decrease in pore size and improved hydrophilicity of the scaffold [37].

The introduction of amino groups onto PCL nanofibers can be achieved with aminolysis reactions. Introducing amino groups has various benefits, including the following: (1) cells/biomolecules can attach to amino group linkers; (2) improved scaffold hydrophilicity; (3) improved water uptake capacity; and (4) neutralized acidic PCL degradation byproducts. Yaseri et al. employed two chemical techniques to modify the surface of PCL nanofibers. They applied hydrolysis (2 M NaOH for 12 h) and aminolysis (2 M HMD/IPA for 12 h) treatments to PCL nanofibers at various concentrations and three predetermined incubation time points. The hydrolysis treatments resulted in the erosion of PCL nanofibers, with an increase in average fiber diameter and reduced average porosity. SEM images of the fibers after the aminolysis treatments suggest less erosion compared to the hydrolysis treatment. With hydrolysis treatments, water contact angles were greatly reduced at the longest incubation time and greatest concentration. With aminolysis treatments, all treatments resulted in reduced and similar water contact angles. Young’s modulus was reduced with both treatments, though the aminolysis impact was greater. Strain was reduced with hydrolysis and increased with aminolysis. Tensile strength was reduced with both treatments, though the hydrolysis impact was greater. Cytocompatibility and cytotoxicity were evaluated using L929 fibroblast cells and an MTT assay. It was also reported that both moderate and prolonged incubation times led to improved cell viability and proliferation [82].

In another study, PCL nanofibers in two groups were enriched with amine or carboxylic acid groups via plasma polymerization, termed COOH-PCL and NH2-PCL nanofibers. A third group enriched PCL nanofibers with amine groups and then immobilized chondroitin sulfate onto their surface, termed CS-PCL nanofibers. The water contact angle of PCL fibers was reduced with plasma treatment and surface immobilization of chondroitin sulfate. The water contact angles were 143 ± 2, 69.1 ± 1.3, 76.5 ± 2.7, and 54.3 ± 1.9 for pristine PCL, COOH-PCL, NH2-PCL, and CS-PCL nanofibers, respectively. Bone marrow stem cells had the greatest survival on chondroitin-sulfate nanofibers, and cartilage formation was best promoted by COOH-PCL nanofibers [75].

Surface functionalization of PCL nanofibers with human collagen type I and fibronectin was reported by Ruocco et al. Following the electrospinning of PCL fibers with an average diameter of 131 ± 39 nm, the scaffolds were immersed in 3,4-dihydroxy-DL-phenylalanine (polyDOPA) and allowed to oxidize and self-polymerize. Then, the scaffolds were immersed in a solution containing human collagen type I and fibronectin. Immunofluorescence images confirm that the PCL scaffolds were coated with both human collagen type I and fibronectin. A BCA test used to quantify the presence of protein available on the surface revealed the presence of the grafted protein coating after 5 days of incubation in PBS. The surface-functionalized PCL scaffolds supported the adhesion, proliferation, and differentiation of cardiac fibroblasts [83]. PGS and PCL were blend-electrospun to covalently immobilize ciliary neurotrophic growth factor (CNTF). First, the electrospun nanofibers were hydrolyzed with NaOH, thereby cleaving the polymers’ ester linkages and forming free carbonyl groups. CNTF was covalently bonded to the carboxylic groups of the polymers to produce biofunctionalized scaffolds [87].

In brief, by increasing porosity and surface wettability with surface modification techniques, scaffolds can better support cell adhesion, bioactivity, and overall tissue integration. Methods such as plasma treatment, aminolysis, and chemical grafting of biomolecules reduce water contact angles and increase hydrophilicity to promote cell adhesion and proliferation. Surface-functionalized scaffolds with biomolecules further improve biocompatibility and mechanical strength.

### 3.3. Surface Coating and Hydrogel Incorporation

The mechanical strength and biocompatibility of porous scaffolds can be supported through interconnected or crosslinked networks [88] and bioactive coatings.

In a multilayer electrospun PCL-printed PCL composite-hydrogel gelatin dip-coating scaffold, it was shown that the water contact angle of PCL scaffolds could be reduced by the self-polymerization of dopamine and further with gelatin dip-coating. The polydopamine (PDA) layer on top of the PCL scaffold serves to (1) increase inter- and intra-molecular interactions and (2) enrich the PCL scaffold with bioactive molecules. It also reduced the water contact angle of the scaffold to 34.4 after 30 s. The water contact angle of the gelatin-coated sample was 0. The mechanical properties of the scaffolds were also measured. Electrospun PCL had a lower Young’s modulus than PCL-PDA gel-coated scaffolds. The strain was measured at the breaking point for electrospun PCL (242 +/− 12%) and increased with PCL-PDA (421 +/− 10%), and even more with gelatin-coating (518 +/− 17%) [89].

Electrospun PLA/PCL nanofibers and PLA/PCL nanofibers coated with bioactive amorphous multi-component SiO_2_-CaO-P_2_O_5_ glass particles using electrospraying were fabricated. Young’s modulus and tensile strength decreased with the incorporation of bioactive particles. The elongation at break was not affected. The presence of the particles reduced the water contact angle by 17.8%. At day 7, cell viability was reduced in PLA/PCL nanofibers coated with bioactive particles but not in uncoated PLA/PCL nanofibers [90]. Similarly, electrospun PCL-chitosan nanofibers were coated with silver and bromelain nanoparticles using a dual electrospray-electrospinning process [91].

Electrospun PCL nanofiber mats were coated with cinnamon oil-loaded chitosan microcapsules. Cinnamon was explored as a component for its anti-inflammatory and antibacterial properties. The cinnamon-loaded chitosan microcapsules and nanofibrous PCL had a synergistic effect on wound healing [92]. PCL and bioactive glass were blend-electrospun to fabricate nanofibrous scaffolds. Then, an ethyl cellulose-based solution with atorvastatin was used to dip-coat the scaffolds. The coating of the scaffolds with ethyl cellulose influenced atorvastatin release. As the concentration of ethyl cellulose increased, the release rate of atorvastatin decreased from a burst release to a gradual linear release profile [85]. Nanohydroxyapatite is a biomaterial that can mimic the minerals present in bone tissue. PCL-gelatin-chitosan nanofibrous scaffolds were fabricated using electrospinning and surface-coated with nanohydroxyapatite. FTIR spectra were consistent with the presence of nanohydroxyapatite on the surface of the treated scaffolds. The treated scaffolds were more supportive of osteoblast cell viability and proliferation than untreated scaffolds [60]. In another study, statin-loaded PCL fibers were dip-coated with silver nanoparticles and collagen [43]. Poly(L-lactide-co-ε-caprolactone) (PLCL) and PCL were blend-electrospun to prepare a nanofibrous membrane and decorated with a fibrin-based coating. The scaffolds were supplemented with human platelet lysate, fibroblast growth factor 2, and vascular endothelial growth factor to create a uniform protein layer. The presence of biomolecules supported cell viability and proliferation [93]. The impact of a gelatin coating on the hydrophilicity and degradation of composite PCL-graphene oxide (GO) nanofibers was investigated by Loyo et al. Water absorption greatly increased from <50% to over 200% with the gelatin coating. Scaffold weight loss in tests of biodegradability was also greatest in gelatin-coated samples (15–25%) compared to uncoated samples (<10%). Additionally, the Young’s modulus of the scaffold increased with GO concentration, while average fiber diameter decreased. The impacts of graphene oxide on mechanical properties were dampened by the gelatin coating, as PCL-GO gelatin-coated scaffolds had lower Young’s modulus, tensile strength, and strain at break than uncoated scaffolds. The gelatin coating decreased cell adhesion in PCL-GO scaffolds [61].

Eugenol is an herbal compound with anti-inflammatory and antioxidant properties derived from essential oils. Eugenol nanoemulsions were mixed with a gelling agent to form nanogels. Separately, PCL-chitosan nanofibers were blend-electrospun. Together, the PCL-chitosan nanofibers and eugenol nanogel achieved the greatest reduction in wound surface area in a Wistar rat model [94]. A hydroxypropyl methylcellulose (HPMC) nanoemulsion was used to encapsulate *Zataria multiflora* in a nanogel. PCL fibers were then coated with the nanogel [41]. PCL and MgO nanoparticles, an antibacterial agent, were blend-electrospun. Then, they were incorporated into a bilayer construct with a collagen gel layer. The presence of PCL within the construct significantly increased its mechanical strength [44]. A bilayer scaffold made of hydrogel and nanofibrous components was fabricated. PCL and gelatin were blend-electrospun onto a collagen-alginate hydrogel and cross-linked [64]. A hydrogel composed of extracellular matrix powder, alginate, and alginate sulfate was fabricated as the two outermost layers of a three-layer composite for tissue engineering. The middle layer was made of PCL and gelatin, blend-electrospun. Sacrificial PVA and gelatin fibers were electrospun concurrently to increase fiber-to-fiber distance [95]. Poly(glycerol sebacate) (PGS)-PCL and methyl methacrylate (PMMA)-PCL scaffolds were fabricated using blend-electrospinning. The scaffolds were then coated with a 150-nm-thick nanocrystalline silver coating. In addition to silver’s antibacterial properties, the thermal stability of the scaffold was enhanced by the silver coating [96].

In summary, crosslinked hydrogels and coatings enhance scaffold mechanical strength and support cell proliferation. Coatings of bioactive molecules, including polydopamine, silver nanoparticles, or bioactive glass particles, both enhance mechanical integrity and reduce water contact angles, thereby promoting cell adhesion and proliferation. However, gelatin coatings dampened the mechanical effects of the scaffold.

### 3.4. 3D Printing

PCL-based nanofibers have been used to enhance the ability of 3D-printed frameworks to support cell seeding.

Plain and drug-loaded PCL scaffolds were tested and compared freestanding or when incorporated into 3D scaffolds with 20% and 50% infills. Compared to freestanding electrospun scaffolds alone, 3D-printed scaffolds had a greater Young’s modulus, with the 50% infill having the greatest Young’s modulus (40.4 MPa) [55].

González Rodríguez et al. fabricated 3D-printed PLA frameworks incorporating PLA-PCL-nanohydroxyapatite electrospun fibers for bone tissue engineering. Various PLA-PCL ratios and concentrations of nanohydroxyapatite were investigated. Of those selected for biological testing, each scaffold was cultured with primary human dermal fibroblasts and analyzed for cell count, viability, proliferation, and morphology. EDS mapping was used for elemental analysis and showed calcium and phosphorus, suggesting the loading of nanohydroxyapatite onto the PLA-PCL nanofibers. Further FTIR analysis suggests the presence of bonds characteristic of nanohydroxyapatite within the scaffold. Thermal analysis suggests the stability of the scaffold within the biological temperature range. SEM images show fibroblast infiltration in the interior of the scaffolds. It was concluded that the greatest cell infiltration was attributed to scaffolds with greater pore sizes and PLA concentration [97]. Highly organized PCL-based scaffolds were fabricated using 3D printing and near-field electrospinning. Hardystonite is a bioceramic that was added to the solution to stimulate adhesion and proliferation [98].

Layered double hydroxide (LDH) nanoparticles are biocompatible agents of tissue regeneration acceleration. Their incorporation into 3D-printed and electrospun PCL-based scaffolds was investigated. Nanofibrous mats made of PCL or PCL-LDH were sandwiched between two 3D-printed circular scaffolds to fabricate hybrid scaffolds. In both wet and dry conditions, PCL-LDH hybrid scaffolds exhibited a higher Young’s modulus than PCL-containing 3D-printed scaffolds and 3D-printed scaffolds alone [89].

In short, PCL-based nanofibers have been used to enhance the mechanical properties and biocompatibility of 3D-printed scaffolds for tissue engineering. Additionally, the incorporation of electrospun PCL blends, such as PLA, nanohydroxyapatite, hardystonite, and LDH, into 3D frameworks can promote cell adhesion and infiltration.

### 3.5. Advanced PCL-Based Scaffold Functionality

Building on the advancements of electrospun PCL scaffolds with natural products and synthetic polymer blends, innovative studies have focused on further enhancing PCL-based scaffold functionality to incorporate improved hydrophilicity and antioxidative properties, as well as conductivity, piezoelectricity, oxygen production, and stimuli responsiveness.

In addition to improving hydrophilicity and cell adherence, additional functionality, such as conductivity, can be achieved. Huner produced nanofibers by electrospinning PCL-conductive polymer blends, which included PCL-poly(m-anthranilic acid) (P3ANA) and PCL-poly(3,4-ethylenedioxythiophene)-poly(styrenesulfonate) (PEDOT). PCL-P3ANA scaffolds had antioxidant activity, and both PCL-P3ANA and PCL-PEDOT nanofibers were tested with MTT assay for toxicity. It was also found with cyclic voltammetry that both formulations were capacitive [99]. In another study, Asadi et al. fabricated a PU-PCL scaffold, a PU-PCL scaffold with soybean oil, and a PU-PCL scaffold with gold nanoparticles and soybean oil. The presence of gold nanoparticles in PU-PCL reduced the water contact angle (76°) compared to PU-PCL-SO (91°). Gold nanoparticles increased the electrical conductivity of the scaffold. Tensile strength and Young’s modulus decreased with soybean oil. No negative effect on cell viability was reported from any of the scaffolds. SEM images show greater NIH-3T3 fibroblast attachment and spreading in the soybean oil and gold nanoparticle-containing scaffolds [100]. MXene is conductive, which gives it potential to be a scaffold material for neural and cardiac applications. MXene was synthesized and immobilized onto electrospun PCL nanofiber membranes. First, the nanofiber membranes were treated with 1 M NaOH for 4 h to improve surface hydrophilicity. The nanofiber membranes were immersed in MXene colloid solution, sonicated, and dried. This process was repeated up to four times. Water contact angle measurements were lowest in scaffolds dipped once and twice in the MXene colloid solution. Conductivity increased with the incorporation of MXene onto the membrane. The morphology and structure of the membranes was analyzed using SEM and energy-dispersive X-ray spectroscopy. Biocompatibility and cytotoxicity were measured using D6P6 human dermal fibroblasts. Both PCL and MXene-PCL membranes showed cell adherence, but twice- and thrice-dipped membranes had greater cell proliferation on day 7 [6].

Piezoelectric materials such as PVDF convert an external force into an electric charge, which can serve to stimulate tissue regeneration. Researchers fabricated nanofibers with various ratios of PCL and PVDF to investigate piezoelectric scaffolds for bone tissue engineering. The average fiber diameter, water contact angle, mechanical strength, and piezoelectric charge coefficient increased with the concentration of PVDF in PVDF-PCL composite fibers. Scaffolds with a PVDF/PCL ratio of 70:30 were determined to be biocompatible [101]. Smart stimuli-responsive materials have also been incorporated into PCL scaffolds, such as liquid crystalline elastomers (LCE). When compared with LCE film, LCE nanofibers, and PCL nanofibers, LCE-PCL nanofibrous scaffolds resulted in higher neuroblast differentiation markers. Additionally, LCE-based scaffolds outperformed PCL-based scaffolds in the expression of other neuroblast differentiation markers. The blending of LCE with PCL improved fiber uniformity. Topological cues induced by the presence of a liquid crystalline phase in the scaffolds on cellular organization were also investigated [102].

The amniotic membrane is an internal layer of the placenta and contains various biomolecules, including growth factors, collagen, and fibronectin. It has low immunogenicity, anti-inflammatory, and antimicrobial properties. Researchers blend-electrospun amniotic membrane-PCL formulations. The composite fibers demonstrated elastoplastic properties. The tensile modulus and elastic strength of the scaffolds increased with amniotic membrane concentration. Amniotic membrane-containing scaffolds showed cell viability and attachment [103].

Plasma-treated PCL-based scaffolds for oxygen generation were fabricated and incorporated into a crosslinked gelatin methacrylate hydrogel. Incorporation of scaffolds within the hydrogel increased scaffold hydrophilicity and swelling capacity in phosphate-buffered saline. The release of oxygen from the scaffolds was measured for 21 days [104]. Oxygen generation and delivery for wound dressings have been achieved by fabricating multilayer scaffolds with peroxides such as sodium percarbonate [79] and calcium peroxide [105]. PCL scaffolds with CaO_2_ nanoparticles immobilized in a gelatin methacrylate hydrogel resulted in oxygen generation [104]. Oxidative stress from reactive oxygen species (ROS) causes damage and injury to tissues. Antioxidants scavenge and neutralize free radicals such as ROS. Ascorbic acid is an antioxidant. It was blend-electrospun at concentrations of 0.15% and 0.3% with PCL to fabricate fibers with the ability to reduce oxidative stress in tissue engineering. The presence of ascorbic acid in electrospun PCL nanofibers resulted in an increase in ultimate tensile strength. The scaffolds were incubated in DPPH and MeOH solutions, and antioxidant activity was measured. The fibers containing ascorbic acid scavenged over 90% of radicals compared to less than 6% in PCL fibers alone. The viability of the scaffolds was determined using a CellTiter Blue Assay of human umbilical endothelial cells cultured for 6 days [106]. Taxifolin is a flavonoid compound and natural product with antioxidant and anti-inflammatory properties. It was loaded onto a cyclodextrin metal-organic framework. Then, the metal-organic framework was electrospun with PCL and PVP. The water contact angle was reduced to 32° after 120 s. It was even lower, at 4°, in cyclodextrin metal-organic frameworks blend-electrospun with PCL and PVP in the absence of taxifolin. The electrospun scaffolds released taxifolin more slowly than the taxifolin-loaded cyclodextrin-metal-organic frameworks alone [107]. Iron-containing metal-organic framework nanozymes were added to an electrospun PCL, gelatin, and glucose composite mesh. As glucose was released from the PCL-based mesh, the nanozymes catalyzed its transition from glucose to antibacterial hydroxyl radicals [108].

A novel light-activated and chemically reactive scaffold was fabricated. PCL with an active ester group for attaching to bioactive amino groups in cells, PCL-NHS, was electrospun with curcumin-loaded manganese nanoparticles [73].

Overall, across PCL-based scaffolds with advanced functionality, cell adherence and proliferation are supported or improved. The incorporation of PVDF, amniotic membrane, and antioxidants such as ascorbic acid supports improved mechanical strength.

## 4. Applications in Tissue Engineering

PCL plays a crucial role in tissue engineering applications and has been widely employed for this specific purpose. In this section, we will focus on different sub-disciplines within the field of tissue engineering where the effectiveness of PCL fibrous mats has been demonstrated to be substantial.

### 4.1. Skin Tissue Engineering

The skin is the body’s largest tissue, which functions as a protective barrier against the external environment and plays a crucial role in thermal regulation and moisture retention, making it essential for human survival. While the skin has a natural ability to regenerate and repair itself, tissue engineering techniques are utilized to create artificial skin substitutes to enhance the healing process of both acute and chronic skin injuries. These injuries are commonly a result of various factors, such as burns, freezing injuries, radiation exposure, surgical interventions, chronic skin ulcers (including venous, pressure, and diabetic foot ulcers), and other dermatological conditions. In order to develop complete skin substitutes, a combination of skin-derived cells (including keratinocytes, dermal fibroblasts, epidermal stem cells, and melanocytes) and non-cutaneous cells (such as inducible pluripotent stem cells (iPSCs), mesenchymal stem cells (MSCs), endothelial cells, and amniotic cells) is utilized as seeding cells. Concurrently, various synthetic and natural biomaterials are carefully chosen and refined for the fabrication of the scaffold. The skin tissue engineering field encounters notable obstacles in creating scaffolds that can accurately replicate the intricate extracellular matrix (ECM) of the skin. The ECM of the skin consists of collagen fibrils organized in a mesh-like configuration, resembling a basket-shaped tissue architecture. It encompasses two separate layers: the epidermis and the dermis, with the epidermis exhibiting a lesser regenerative capacity in contrast to that of the dermis. Consequently, the objective of tissue engineering is to not only facilitate wound healing but also stimulate the regeneration of both skin layers in a manner that closely mimics their inherent composition and structure.

As mentioned earlier, the incorporation of various materials with polymer fibers is utilized for skin tissue engineering. PCL is one such polymer that has been utilized widely in this field. Various formulations with PCL fiber scaffolds have been utilized for skin tissue engineering. PCL is used individually and combined with natural and synthetic materials for skin tissue engineering applications [98]. Drug-loaded PCL has been studied for its potential in wound dressing applications. In a recent study, antibacterial porous biocompatible PCL fiber scaffolds loaded with chloramphenicol (CAM) were created for the purpose of healing infected wounds [109]. The morphology, structure, and elasticity of PCL fiber scaffolds electrospun using various solvent systems (including tetrahydrofuran (THF), dimethyl sulfoxide (DMSO), acetic acid (AA), and formic acid (FA)) exhibit notable differences, influencing their interactions with both bacterial and eukaryotic cells. While electrospinning enables the production of diverse structures, this study uniquely highlights the impact of surface pores on drug-loaded fibers regarding the attachment of eukaryotic and bacterial cells. Specifically, porous CAM-loaded microfiber scaffolds (THF/DMSO solvent system) demonstrate enhanced fibroblast attachment and growth compared to nonporous microfiber (THF/DMSO solvent system) and nanofiber scaffolds (AA/FA solvent system). Additionally, the porous CAM-loaded microfiber scaffolds exhibit superior antibiofilm activity against *E. coli*. This research underscores the significance of considering the surface porosity of individual fibers alongside fiber diameter, as these factors can influence drug release and mechanical properties.

One of the most utilized materials in this field is gelatin, which has shown great benefit when combined with PCL [99,110,111]. Blend, coaxial, and uniaxial electrospinning techniques have been utilized to study their effect on the mechanical strength of gel/PCL composites. Gelatine and PCL individually showed weak mechanical strength; however, their composites exhibit higher mechanical strength. Among the composites, blend systems possess the highest mechanical strength [112]. Single-layer wound dressings are insufficient to address all clinical requirements because of their distinct characteristics and limitations. To effectively manage the complexities associated with wound care, it is essential to carefully choose appropriate dressings. The incorporation of collagen with nanofibers (NFs) serves to augment the characteristics of dressings, thereby enhancing cellular interactions. Multi-layered nanofibrous systems, especially those incorporating polycaprolactone (PCL), exhibit superior mechanical properties and extended release of therapeutic agents. Hajati Ziabari et al. reported a study in which four variations of nanofibrous bilayer mats (NBMs) were produced through the electrospinning technique, consisting of an active layer comprising a blend of PCL and collagen with and without Malva sylvestris (MS) extract and an inactive layer solely containing PCL. The fabricated bilayers included neat PCL/PCL-Collagen (PCLC), PCL/PCLC-5% MS, PCL/PCLC-10% MS, and PCL/PCLC-15% MS [113]. The effective retrieval of natural compounds from mulberry silk (MS) through maceration was successfully demonstrated, reaching an equilibrium state within 48 h. The introduction of MS extract had a negligible effect on fiber diameter, maintaining consistency. Additionally, the inclusion of MS extract led to improved ultimate tensile strength, showcasing its mechanical appropriateness for supporting tissues during the wound healing process. The produced NBMs exhibited exceptional blood hemocompatibility, with hemolysis rates remaining below the critical threshold of 2%. Noteworthy reductions in prothrombin time (PT) and activated partial thromboplastin time (APTT) values were observed, suggesting their potential for enhancing blood coagulation. The NBMs demonstrated promising effectiveness in the management of exudates at the wound site, which is a critical component of wound care. Research on cell attachment and viability indicated that the NBMs successfully replicate biological conditions, underscoring their potential for use in wound healing treatments. In summary, the findings suggest that NBMs hold promise for enhancing wound healing processes.

**Figure 9:**
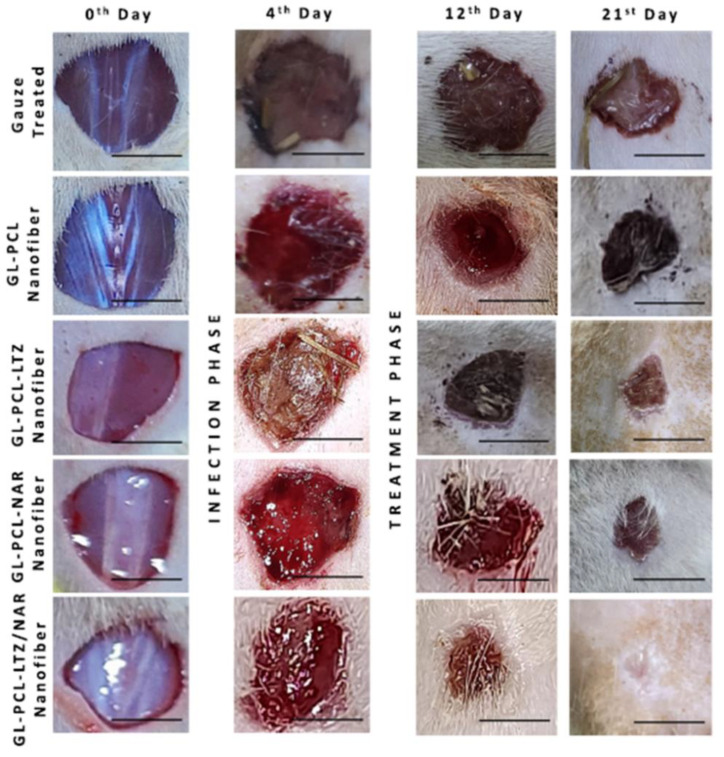
In vivo wound healing of gelatine-coated PCL nanofibers as well as gelatine-coated drug-loaded PCL nanofibers [120].

Along with fiber porosity and fiber diameter, the fiber-to-fiber distance in such systems plays a significant role in designing scaffolds for dermal-epidermal scaffolds [114]. In a recent study by Gao C. et al., a multilayer undulating scaffold to mimic rete ridges was fabricated with electrospun PCL (PCL nanofiber and microfiber composite structures), PCL-PDA (polydopamine-coated PCL), and PCL-PDA-Gel (gelatin-coated PCL-PDA) [84]. The uniaxial electrospinning technique was utilized to prepare the PCL nanofibers, followed by the 3D printing of PCL microfibers on the nanofiber mat. The PDA was coated onto the PCL nanofiber/microfiber composite. Finally, the PDA-coated PCL structures were drafted with 5% (*w*/*v*) gelatin. The average diameter of PCL nanofibers was 575 ± 133 nm. The effect of fiber-to-fiber distance was studied for distances of 100, 200, 300, and 400 µm for the PDA-printed layer. Various techniques, including live and dead staining, cell counting kit-8 (CCK-8), histology using haematoxylin and eosin (HE) staining, and immunofluorescence with primary and secondary antibodies, were employed to assess the viability, metabolic activity, and differentiation of skin cells during in vitro culturing. The in vitro study showed that the greatest proliferation was found at a 100 µm fiber-to-fiber distance relative to other fiber-to-fiber distances. It was concluded that decreasing the distance between fibers was more favorable for cell viability, proliferation, and differentiation. In another innovative study by Rezvani Ghomi E. et al., biocompatible tunable scaffolds comprising PCL/gel/ε-polylysine (ε-PL) were prepared to study their potential in wound dressing applications [115]. Scaffolds were prepared by 3D-printed PCL micromeshes coated with electrospun PCL/gel/(ε-PL) nanofibrous mats bearing nanopores, followed by crosslinking using a UV source to strengthen the final scaffold. The nanofiber diameter before and after crosslinking was found to be 266 ± 83 nm and 402 ± 166 nm, respectively. These measurements closely aligned with the dimensions of the collagen monomers in the skin ECM fibers [116]. The innovative bimodal scaffold showed excellent mechanical strength while possessing micro- and nanopores that mimic the human skin. The scaffolds exhibited strong cytocompatibility and supported the proliferation of host cells. Hemolysis testing indicated that the results were in accordance with established international standards for biomaterials. Evaluation using the live/dead assay with fibroblasts and HaCaT cells revealed exclusive blue fluorescence following a 24-h incubation with 3D-printed PCL micromesh and bimodal scaffold, indicating cell viability. In contrast, the positive control displayed red fluorescence upon staining with propidium iodide, signifying cell death post-treatment. The prepared scaffolds showed a great potential for wound dressing/skin tissue engineering applications. Combining PCL/Gel with graphene oxide (GO) and other materials has proven to be effective for chronic wound healing. The reparation of conductive bilayer scaffold wound healing applications was reported by Song Z. et al. [117]. Biomimetic bilayer skin scaffolds were prepared by combining PCL/Gel fiber mats with conductive non-woven layers composed of Chitosan (Chi)@rGO (reduced graphene oxide). The fibrous scaffold mimicked the epidermis, while the loose non-woven conductive layer mimicked the dermis layer. The compact electrospinning membrane exhibited an appropriate fiber diameter conducive to the growth and attachment of keratinocytes, thereby promoting the re-epithelialization process of wounds. The porous structure of chitosan non-woven fabrics facilitated the penetration of fibroblasts, while the incorporation of reduced graphene oxide (rGO) imparted electrical conductivity to the fabric, improving intercellular electrical signal transmission. This, in turn, stimulated fibroblast activity and migration. Skin scaffolds must possess effective exudate absorption capabilities to mitigate the accumulation of exudate within wounds. The skin scaffolds composed of PCL/Gelatin-Chi@rGO developed in this study demonstrated the ability to efficiently inhibit the excessive accumulation of wound exudate, which promoted the process of wound healing. Following the assembly of the scaffold, a single-step chemical cross-linking process utilizing glutaraldehyde was employed to enhance the structural integrity of the PCL/gelatin nanofiber membrane in aqueous conditions. This treatment also reinforced the adhesive strength of the dual-layered structure. The textile-derived biomimetic artificial skin scaffold developed in this investigation exhibits favorable mechanical characteristics suitable for practical applications, alongside exceptional biological functionalities. This scaffold holds significance particularly for wounds with limited regenerative capabilities, such as diabetic wounds, deep trauma wounds, and extensive area wounds. The incorporation of GO with PCL/Gelatine electrospun fibers is also studied for its antimicrobial activity. In a recent study by Hamdan N. et al., PCL/gel/GO electrospun nanofibers (PGO) are modified through plasma treatment with the monomeric groups diallylamine (PGO-M1), acrylic acid (PGO-M2), and tert-butyl acrylate (PGO-M3) to study their antibacterial activity against Staphylococcus aureus (*S. aureus*) and Escherichia coli (*E. coli*) [118]. The surface hydrophilicity, which is closely linked to the chemical functionalization of nanofibers, played a significant role in influencing the in vitro cell behavior and antibacterial properties. Specifically, a higher growth of *S. aureus* colonies was observed on PGO-M3 surfaces compared to PGO-M1 and PGO-M2 surfaces. Conversely, L929 cells exhibited enhanced adhesion and proliferation on PGO-M1 surfaces. Furthermore, the antimicrobial activity against *E. coli* bacteria was found to be the highest on PGO-M1 surfaces, followed by PGO-M2 and PGO-M3 surfaces. The chemical surface functionalization led to selective interaction between the functional groups and different bacterial populations. Gelatine-coating on PCL nanofibers is another technique that has proven to enhance the wettability and bioadhesion of the scaffolds and improve their wound healing performance [119]. A similar technique has been utilized by Manjit M. et al., who reported the in vivo wound healing efficiency of drug-loaded PCL nanofibrous mats coated with gelatine [120]. Different formulations were tested to analyze their scaffolds’ in vitro cytocompatibility and in vivo drug release as well as wound healing capability: gelatin-coated PCL (GL-PCL) scaffold, gelatin-coated Luliconazole (LTZ)-loaded PCL scaffold (GL-PCL-LTZ), gelatin-coated Naringenin (NAR)-loaded PCL scaffold (GL-PCL-NAR), and gelatin-coated NAR- and LTZ-loaded scaffold (GL-PCL-NAR/LTZ). The co-axial electrospinning technique was employed to prepare core-shell (gel as shell, and PCL/drug-loaded PCL as core) fibrous mats. The average fiber diameter for each formulation was found to be 213.35 ± 52.55 nm, 341.48 ± 115.56 nm, 354.99 ± 97.33 nm, and 379.67 ± 166.62 nm, respectively. The GL-PCL-LTZ and GL-PCL-LTZ/NAR demonstrated significant efficacy in eliminating *C. albicans* and *C. tropicalis*. In vitro cytocompatibility studies indicate that all nanofibers effectively enhance cellular proliferation owing to the three-dimensional morphological structure of the nanofiber matrix. In vivo wound closure investigations (Figure 9) reveal that GL-PCL-LTZ/NAR notably enhances the wound-healing capacity of GL-PCL nanofibers. This enhancement may be attributed to the ability of GL-PCL-LTZ/NAR to maintain a sterile environment and improve blood circulation in the vicinity of the wound site.

**Figure 10:**
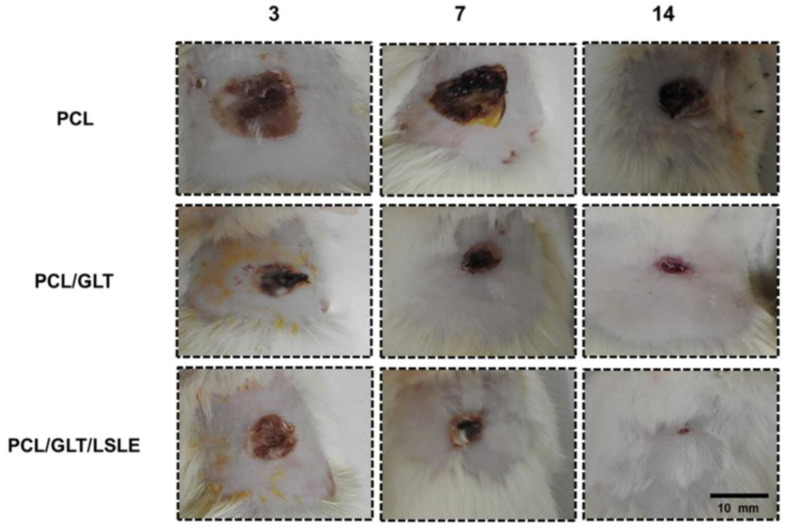
Burn wound healing progress at days 3, 7, and 14 for PCL, PCL/GLT, and PCL/GLT/LSLE electrospun fibrous mats [121].

The utilization of green materials in the fields of biomedical and bioengineering has experienced notable growth in recent times. In one such study, the utilization of egg white (EW) was blended with gel/PCL fibrous mats to analyze its potential in skin tissue engineering. This study presents an innovative nanofibrous scaffold designed for skin tissue engineering, which integrates gel and EW as affordable and easily accessible materials, demonstrating significant promise for a range of biological uses [70]. Gel/PCL nanofibers blended with 10% EW has shown significant improvement in efficiency of fibroblast culture. The fibroblasts play a significant role in the wound healing process; therefore, their enhanced efficiency is one of the most important factors that needs to be considered while designing a wound dressing. Recently, a few studies have highlighted the potential of using fly maggots, specifically those of the green blowfly (*Lucilia sericata* or *L. sericata*) species, for healing skin wounds. In one such innovative study, in vivo burn wound healing was studied. *L. sericata* larva extract (LSLE) was blended with gelatin (GLT)/PCL nanofibers to study their wound-healing efficiency [121]. The nanofibrous mat composed of PCL/GLT/LSLE exhibited a porous structure characterized by well-branched features, with an average diameter measuring 500.2 ± 20.46 nm. The concentration of LSLE has a direct impact on reducing the production of pro-inflammatory cytokines, such as TNF-alpha, IL-12, and MIF, while simultaneously increasing the production of anti-inflammatory cytokines, such as IL-10, by inflammatory cells. Furthermore, LSLE exhibits the capacity to impede the complement system. This modulation of the inflammatory response contributes to expediting the process of wound healing. The in vivo analysis of the scaffolds showed that animal wounds treated with PCL/GLT/LSLE showed improved granulation and re-epithelialization outcomes (Figure 10). Additionally, LSLE increased the expression of extracellular matrix elements, including collagen type I. The enhanced wound-healing capabilities of the PCL/GLT/LSLE matrix observed in the experimental wound model in this investigation may be ascribed to the bioactive constituents present in LSLE, which exert influence across all stages of the wound healing process.

Similarly, in another study, a natural antibacterial agent, tannic acid (TA), blended with PCL nanofibers was analyzed for its potential in wound dressing applications [122]. The presence of TA with PCL enhanced the morphology of the fibers due to the increased conductivity of the spinning solution. The incorporation of TA resulted in increased hydrophilicity and water absorption of the scaffolds, promoting wound healing. Furthermore, a rapid initial release of TA within the first hour followed by a gradual and controlled release was noted. The quantity of TA released from the PCL/TA (9:1) nanofiber scaffold within a 24-h period appeared to be effective in suppressing bacterial proliferation. The *Moreinga olefeira* (*M. oleifera*) plant is a herbal medicine traditionally used in wound healing. Sadia M. et al. analyzed M. oleifera-loaded PCL fibers for their wound healing potential [75]. In this research investigation, extracts derived from Moringa oleifera leaves were integrated into an electrospun PCL nanofibrous membrane at different concentrations (19 wt%, 39 wt%, and 59 wt%). Additionally, a standard proportion of 1 wt% silver nanoparticles (AgNPs) was introduced to enhance the overall performance of the membrane. These membranes exhibited a higher efficacy in eradicating the Gram-positive bacterium *S. aureus* compared to the Gram-negative bacterium *E. coli* by releasing antibacterial agents and employing antibacterial mechanisms. The incorporation of methanolic extract derived from M. oleifera and silver nanoparticles (AgNPs) resulted in an enhancement of the platelet coagulation properties of the PCL membranes, thereby categorizing these membranes as hemocompatible materials.

The emerging Janus membranes featuring a hydrophobic–hydrophilic interface, such as fiber-based, sponge-based, hydrogel-based, and polymer-based dressings with asymmetric wetting properties, have been shown to enhance the removal of excess biofluid surrounding wounds by facilitating unidirectional water transfer from the hydrophobic side in contact with the skin to the hydrophilic side facing outward. This mechanism effectively inhibits the creation of a conducive environment for bacterial proliferation. PCL has been explored for the Janus fibrous structures’ hydrophobic layer. In a recent study, PCL/gel fiber mat was used to prepare the hydrophilic layer, while the silver-doped piezoelectric polyvinylidene fluoride (PVDF) made the hydrophobic layer to create a Janus fibrous structure [123]. In the hydrophobic layer, the incorporation of silver nanoparticles (Ag NPs) not only enhanced the antibacterial properties of the Janus film against both Gram-negative and Gram-positive bacteria but also strengthened the piezoelectric characteristics of the nanofibers. The piezoelectric properties, along with the well-aligned nanofibers, facilitated the directed migration of fibroblasts, thereby promoting accelerated wound healing. The effectiveness of the Janus fiber films in wound healing was demonstrated in a full-skin wound model infected with *S. aureus*, showing efficient wound closure, bacterial infection control, decreased levels of pro-inflammatory cytokines, and enhanced processes of angiogenesis and collagen formation. The primary benefit of Janus membranes lies in their capacity to facilitate wound healing by efficiently draining wound exudate, owing to their asymmetric wettability.

**Figure 11:**
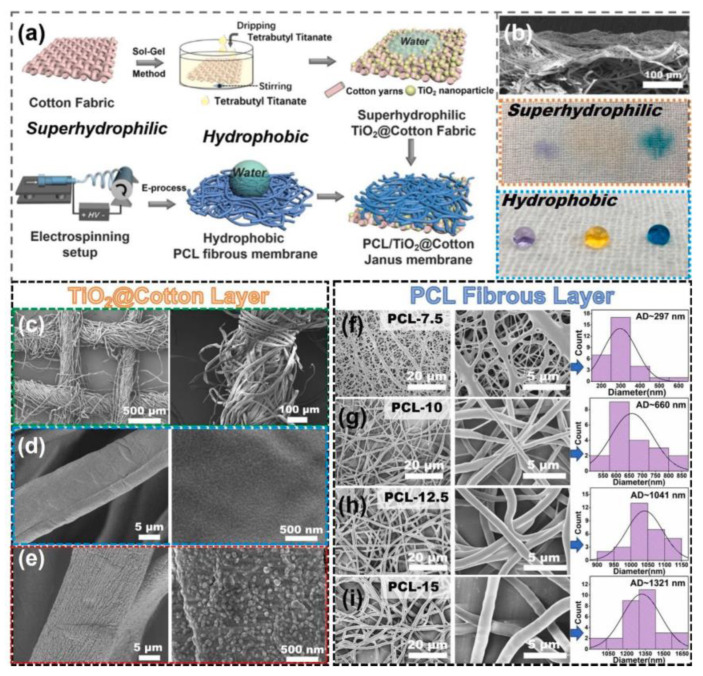
(**a**) Schematic of the fabrication process of the PCL/TiO_2_@Cotton Janus membrane. (**b**) SEM image of the cross-section of the Janus membrane (**top**). Photographs of water droplets (~30 μL) on the TiO_2_@Cotton layer (**middle**) and the reversed PCL fibrous layer (**bottom**). Water droplets were colored by dyes (Acid Violet 7, Sunset Yellow FCF, Alkali Blue 70). (**c**) SEM images of pristine cotton fabric and its partially enlarged cotton yarn; (**d**) single cotton yarn and its partial enlarged image; (**e**) TiO_2_@Cotton yarn and its partial enlarged image. (**f**–**i**) SEM images, partial enlarged SEM images, and statistically average fiber diameters corresponding to PCL-7.5, PCL-10, PCL-12.5, and PCL-15, respectively [126].

In a recent study, directional liquid transport was analyzed to study its effect on wound healing [124]. The superhydrophilic layer was composed of a TiO_2_-anchored cotton gauze dressing (TiO_2_@Cotton), while PCL (7.5 wt%, 10 wt%, 12.5 wt%, and 15 wt%) nanofibers were used to design the hydrophobic layer. Figure 11 shows the preparation process and morphology of both layers. This design demonstrated unique directional liquid transport capabilities due to the combined effects of varying wettability, the antibacterial properties from TiO_2_, and the biocompatibility of PCL. By effectively pumping wound exudate away from open wounds and maintaining a dry environment for healing, the Janus membrane shows promise in promoting wound healing.

In another study, a PCL mesh-like structure was utilized as a hydrophobic layer, and PEG/PCL electrospun nanofibers loaded with deferoxamine (DFO) made the outer hydrophilic layer [125]. The resultant Janus membrane was analyzed for its potential in diabetic wound healing. The results obtained from in vitro experiments demonstrated that the Janus dressing exhibited favorable biocompatibility. The mesh-like structure of the inner surface was found to be conducive to fibroblast attachment and proliferation, while the controlled release of DFO notably improved tube formation. Findings from in vivo studies using a diabetic full-thickness cutaneous wound model indicated that the Janus dressing established a distinctive microenvironment that expedited wound healing by facilitating efficient drainage of biofluids and promoting the angiogenesis process. Effective management of biofluids in wounds plays a critical role in facilitating the healing process. Presently, conventional wound dressings that are specifically engineered for facilitating directional water transport encounter difficulties when used to treat wounds located in joint areas. To address this challenge, Yang L. et al. reported a study in which a hybrid manufacturing technique was suggested to create a bilayer membrane with enhanced durability, superior directional water transport abilities, and antibacterial characteristics. This method combines solution electrospinning (SE) and melt electrowriting (MEW) processes to prepare a Janus membrane [126]. This novel method offers exceptional stretchability, directional water transport, and antibacterial properties. Research has shown that the structure of the PCL MEW scaffolds significantly influences their mechanical characteristics. In comparison to other structures, the knit-like scaffold demonstrated a unique J-shaped behavior in the force–strain curve during initial stretching, resembling the deformable nature of skin. Furthermore, it exhibited notable elastic recovery, making it well suited for use as the hydrophobic layer in composite dressings. The SBS nonwoven fabric, generated through electrospinning and treated with O_2_ plasma, displayed good hydrophilicity and elasticity, functioning as the hydrophilic layer. Additionally, tests for antibacterial activity and moisture management confirmed that the bilayer membrane exhibited effective antibacterial properties and directional water transport capabilities.

In the last decade, three-dimensional (3D) bioprinting has become a new and innovative technological method for fabricating intricate structures by depositing bioinks containing cells in a layer-by-layer fashion. This technique has been employed to create wound dressings. Three-dimensional printing encompasses several methods, such as stereolithography, micro-extrusion, laser-assisted, and inkjet printing. By carefully formulating bioinks using a range of materials, it becomes possible to bioprint structures of diverse architectures that exhibit robust mechanical stability. Bio-inks consist of natural substances such as hyaluronic acid, alginate, collagen, chitosan, chitin, cellulose, and thrombin, as well as synthetic materials such as ε-caprolactone, PLA, PGA, PU, polycarbonates, trimethylene carbonate, polyethylene glycol methacrylate, and poly(propylene fumarate) [127]. In a recent study, a core/shell fiber scaffold was created by applying a uniform layer of polycaprolactone (PCL) onto 3D-printed alginate-gelatin hydrogel scaffolds [128]. The PCL coatings decreased the unrestricted release of drugs from the core gels. Following this, polydopamine (PDA) was applied to the gelatine (gel)/PCL core/shell scaffolds, enhancing their photothermal properties. As a result, near-infrared (NIR) laser activation enabled controlled drug release when needed, facilitated by the thermally induced sol-gel transition of the core gels. The gel/PCL/PDA core/shell fiber scaffolds served as a foundation for targeted cancer treatment and the enhancement of tissue regeneration, particularly in the context of wound healing.

A few research reports also suggest the high potential of PCL when blended with other polymers and materials. In one such study, Sc_2_O_3_-MgO particles were synthesized and incorporated into electrospun PCL/PVP nanofiber membranes [30]. The addition of 2.0 wt% Sc_2_O_3_-MgO resulted in a membrane consisting of uniform fibers embedded with white nanoparticles of an average size of 256.2 nm. Following a 24-h period, the membrane exhibited effective bacteriostatic activity against *E. coli*, achieving a bacteriostatic rate of 100%. These findings suggest that the developed electrospun nanofiber membranes possessing notable antibacterial properties could serve as a viable material for tissue engineering applications, aiding in the prevention of bacterial proliferation at wound sites and addressing postoperative infection concerns.

### 4.2. Bone Regeneration

Bone is a highly active tissue that experiences continuous remodeling over the course of an individual’s lifespan, maintaining a delicate equilibrium between the degradation of old mineralized bone and the generation of new bone structures. This process of bone remodeling or repair is facilitated by specialized osteogenic cells, namely osteoblasts, osteocytes, osteoclasts, and bone lining cells, each with distinct roles and functions.

*Osteoblasts* are cells responsible for the formation of bone tissue. They produce a collagen matrix and participate in the synthesis of various proteins, including osteopontin and osteocalcin. Following the process of active bone formation, osteoblasts are believed to undergo one of three possible outcomes: firstly, they may transition into a dormant state as lining cells situated on the bone surface; secondly, they may experience programmed cell death, known as apoptosis; and thirdly, they may embed within their own osteoid and transform into osteocytes.

*Osteocytes* are mature osteoblast cells that possess the ability to transmit signals in response to mechanical stress and strain. They represent the predominant cell type within bone tissue and are essential for the maintenance of skeletal homeostasis in adult individuals. Osteocytes serve a pivotal role in orchestrating bone remodeling by modulating the activities of both osteoblasts and osteoclasts.

*Osteoclasts* play a crucial role in the resorption and elimination of aged bone cells, functioning as cells responsible for bone resorption. These specialized cells originate from hematopoietic precursors of monocyte or macrophage lineage. The cells of the osteoblast lineage facilitate the effects of factors that promote bone resorption, leading to the differentiation of osteoclasts from their precursor cells.

*Bone lining cells* are responsible for regulating the absorption and release of minerals in bone tissue. The maintenance of bone tissue relies on a delicate equilibrium between bone formation and bone resorption processes. Cells derived from the osteoblast lineage, such as osteoblasts, osteocytes, and bone lining cells, contribute to bone formation, whereas osteoclasts are responsible for bone resorption.

Synthetic bone tissue scaffolds are expected to replicate the structures and functions of natural bone while possessing favorable mechanical properties and biocompatibility. Therefore, it is essential to understand the physicochemical architecture of native bone, as well as relevant biomechanical and biochemical properties, to inform the selection and utilization of optimal biomaterials for constructing bone tissue scaffolds. Bone is a complex and specialized connective tissue consisting of approximately 70% inorganic constituents, primarily hydroxyapatite (HA), nearly 20% organic constituents, and 10% water. The most abundant organic constituent is type I collagen, making up around 90% of the organic components, which not only anchors HA crystals but also promotes cellular attachment through its abundant RGD (Arg-Gly-Asp tripeptide sequence) residues. Bone serves crucial functions in human physiology, including providing support for the body and vital organs, facilitating movement, blood production, mineral storage, maintaining homeostasis, and serving as a reservoir for various progenitor cells, such as mesenchymal and hematopoietic stem cells.

**Figure 12:**
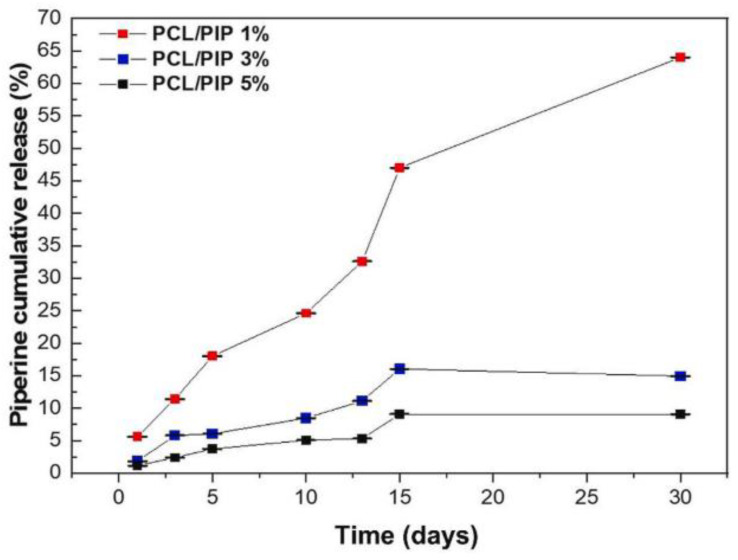
Cumulative drug release profile of different PIP-loaded PCL formulations [129].

Extensive research has been conducted on polymeric drug scaffolds in the field of orthopedic surgery, as they can release therapeutic agents in a controlled and targeted manner at the desired location. PCL has been utilized for controlled drug release for bone regeneration. In a recent study by Oliveira S. et al., electrospun piperine (PIP)-loaded PCL (PIP/PCL) scaffolds have been analyzed for their drug delivery characteristics. Different concentrations of PIP were analyzed for these electrospun scaffolds, i.e., PCL, PCL/PIP 1%, PCL/PIP 3%, and PCL/PIP 5% [129]. The average diameters for each scaffold PCL, PCL/PIP 1%, PCL/PIP 3%, PCL/PIP 5% were measured. The release of piperine exhibited significant levels after a period of 15 days. Consequently, at the 30-day mark, the PCL/PIP 1% scaffold demonstrated a release of 64% ± 0.00062, whereas the PCL/PIP 3% and PCL/PIP 5% scaffolds exhibited lower release percentages of 16.06 ± 0.00012 and 9.08% ± 0.00031, respectively, and the average diameters were measured to be 0.0015 ± 0.002 μm, 1.08 ± 0.007 μm, 1.09 ± 0.02 μm, and 0.59 ± 0.016 μm, respectively. Figure 12 shows the cumulative drug release of the prepared formulations. Additionally, PCL/PIP 1% and PCL/PIP 5% demonstrated a notable bone restructuring trend in contrast to the remaining groups.

PCL/gelatine composites have been utilized for bone tissue engineering due to the good mechanical strength of PCL and the high biocompatibility of gelatine. Drawing inspiration from the natural periosteum’s structure and function, Zhao X. et al. have developed an electrospun biomimetic periosteum aimed at enhancing bone regeneration through the activation of innate bone healing processes [130]. This biomimetic periosteum consists of a two-layered design with an asymmetric configuration. The outer layer is made of aligned electrospun poly(ε-caprolactone)/gelatin/deferoxamine (PCL/GEL/DFO) to replicate the fibrous outer layer of the periosteum, while the inner layer comprises a random coaxial electrospun PCL/GEL/aspirin (ASP) shell and a PCL/silicon nanoparticles (SiNPs) core to mimic the cambial inner layer. This bilayer structure enables the controlled release of ASP, DFO, and SiNPs, thereby regulating the inflammatory, angiogenic, and osteogenic phases of bone repair in a precise manner. The biomimetic periosteum demonstrated superior mechanical characteristics, high biocompatibility, and the ability to release ASP, DFO, and SiNPs in a controlled manner. Notably, the outer layer of aligned fibers effectively prevents the invasion of soft tissues and encourages the formation of new blood vessels, while the inner layer possesses anti-inflammatory, antioxidant, and activities that promote the growth and specialization of bone-forming cells.

In experiments conducted on living organisms, it was observed that the biomimetic periosteum, which releases multiple agents, facilitated successful bone regeneration in cranial defects in rats by enhancing the bone repair process. In summary, the incorporation of multiple agents into electrospun bilayer membranes with asymmetric properties for different stages of bone healing has led to the creation of a periosteum that mimics the structure and function of natural periosteum. The utilization of guided bone regeneration (GBR) technology has been widespread in addressing alveolar bone defects. Nevertheless, current GBR membranes exhibit significant deficiencies in terms of their biological efficacy and regenerative capacity. To address this challenge, Xu J. et al. fabricated a bilayer composite-guided bone regeneration (GBR) membrane using high-speed electrospinning technology, featuring an oriented structure composed of silk fibroin (SF)/bioactive glass (BG) and SF/PCL [131]. This membrane serves the dual purposes of promoting bone regeneration and acting as a barrier. The SF/PCL membrane, characterized by its relatively high density, functioned as a barrier to separate bone defects from surrounding soft tissues. In contrast, the SF/BG membrane was employed to enhance the osteogenic characteristics of the bone defect region. The incorporation of polycaprolactone (PCL) has been shown to enhance the tensile characteristics of the SF/PCL layer. Furthermore, the integration of SF/PCL layer, along with the membrane, has been demonstrated to enhance the mechanical properties of a singular material, thereby facilitating bone tissue regeneration. The oriented SF/BG@SF/PCL has shown excellent biocompatibility, which was analyzed through in vitro studies. Moreover, the SF/BG composite fiber membrane featuring an oriented structure exhibited a pronounced osteogenic impact, fostering a conducive environment for the osteogenic differentiation of bone marrow mesenchymal stem cells (BMSCs). This suggests that the microstructural characteristics of the material surface play a crucial role in modulating the biological responses of cells. The morphological organization of cells in the oriented SF/BG group exhibited greater clarity in comparison to the SF/BG group, thereby facilitating the augmentation of their biological activity and the efficient stimulation of osteogenic cell differentiation.

In another study, a core-shell GBR membrane composed of PCL/chitosan(CS)/polyvinyl alcohol (PVA) was fabricated with varying amounts of resveratrol (RSV), enhancing its osteogenic induction ability compared to a standard GBR membrane [132]. The study conducted on the release of RSV in vitro showed that the membranes maintained a consistent RSV release pattern over a span of 15 days. Additionally, the amount of RSV released increased from 39.68 ± 2.09 μg to 65.8 ± 2.91 μg as the RSV content in the core layer of the GBR membranes rose from 0.1% to 0.5% (*w*/*v*). Notably, the PCL/CS/PVA GBR membrane containing 0.5% RSV demonstrated the most effective sustained and controlled release of RSV, leading to enhanced osteogenic differentiation of pre-osteoblasts in vitro and bone regeneration in vivo. The histological analysis revealed that defect sites treated with the GBR membrane loaded with 0.5% RSV exhibited newly formed bone tissues with 82.46 ± 9.86% BV/TV and 0.70 ± 0.07 gcm^−3^ BMD after 12 weeks, showcasing the highest values compared to the other groups. In conclusion, the PCL/CS/PVA GBR membrane loaded with 0.5% RSV shows promising potential for bone regeneration.

The periodontal tissue is composed of cementum, periodontal ligament, alveolar bone, and gingiva, forming a complex three-dimensional structure that plays a vital role in preserving the proper alignment and functionality of the teeth. The successful regeneration of this intricate and organized tissue structure hinges on the accurate simulation of its highly layered composition. Hua W. et al. reported the successful production of electrospun nanofibrous mats made from PLA–PCL with freeze-dried chitosan, featuring a directionally arranged microporous channel structure, utilizing the electrospinning, directional freeze-drying, and cross-linking techniques [133]. Additionally, nanoparticles were incorporated to load three distinct growth factors onto the target layer, resulting in the construction of triphasic scaffolds that closely mimic the physiological periodontal tissue. The scaffolds were confirmed to have advantageous mechanical characteristics, outstanding biocompatibility, and minimal cytotoxicity during in vitro testing. Each section of the structure containing growth factors was found to enhance the activation of genes related to osteogenesis, periodontal ligament, and cementogenesis in periodontal ligament stem cells (PDLSCs). In an in vivo setting, the biomimetic scaffolds infused with growth factors were also proven to effectively treat periodontal defects in rats by stimulating the development of a healthy periodontium. Repairing different types of periodontal defects requires a strategic approach that involves the use of a bio-scaffold capable of guiding local stem cells through the processes of migration, adhesion, proliferation, and differentiation.

In this context, Liang C. et al. reported a novel approach involving the integration of a cell-specific decellularized extracellular matrix (ECM) with a biomimetic electrospinning scaffold to facilitate the regeneration of severely damaged periodontal tissue [134]. The researchers fabricated a gelatin/PCL scaffold featuring a triple-layered structure (TLS) with an aligned-random-aligned configuration. This TLS comprised aligned fibers on the PDL-side and bone-side surfaces, while the middle layer consisted of randomized fibers, creating a grid-like porous structure. The structure was composed of separate regenerative hubs designed to promote the regeneration of the periodontal ligament (PDL) and alveolar bone. The resulting scaffold, known as Bi-ECM-TLS, functioned as both a barrier film and a stimulator for regeneration by offering signals for the regeneration of the PDL and alveolar bone (AB). This design not only enhanced the scaffold’s strength but also enabled it to effectively resist forces from multiple directions. The controlled degradation of the TLS was essential for providing structural support during the initial stages of tissue regeneration while also allowing for gradual replacement by the newly formed tissues over time. Guided tissue regeneration (GTR) and bone tissue regeneration (BTR) are surgical procedures that involve the utilization of a membrane to inhibit epithelial growth within a defect, thereby creating an environment conducive for periodontal and bone cells, including stem cells, to regenerate and repair the damaged tissues. The efficacy of these treatments is often compromised by the presence of local bacterial colonization around the membrane and its rapid degradation, leading to postoperative infections and premature membrane rupture, which hinders the regeneration process.

Prado-Prone G. investigated the antimicrobial and bone-forming characteristics of electrospun membranes made from PCL-gelatin (PCL-G) that have been enhanced with zinc oxide nanoparticles (ZnO-NPs) [135]. The PCL-G membranes were subjected to modification with varying concentrations of ZnO-NPs, specifically 1%, 3%, and 6% (*w*/*w*). The outcome of this modification was a notable decrease in both planktonic and biofilm formation of four bacteria strains that are clinically relevant, namely *A. actinomycetemcomitans* serotype b, *P. gingivalis*, *E. coli*, and *S. epidermidis*. Furthermore, these modified membranes exhibited suitable mechanical properties and biodegradation rates, making them potentially suitable for use in clinical treatments. It is worth mentioning that the membranes treated with the lowest concentration of ZnO-NPs (1% *w*/*w*) demonstrated the ability to stimulate the production of osteoblast markers and calcium deposits in human bone marrow-derived mesenchymal stem cells (BM-MSC). Additionally, they were found to be biocompatible with human osteoblast cells (hFOB). These findings indicate that the PCL-G membranes containing 1% *w*/*w* of ZnO-NPs have a high potential for use in GTR/BTR treatments, as they were the most effective in terms of antibacterial effectiveness at a lower NP concentration, while also creating a favorable cellular microenvironment for bone growth.

As mentioned earlier, the incorporation of graphene oxide in PCL-based scaffolds has been studied extensively for tissue engineering applications. Graphene oxide (GO) has significant potential as an additive in polymer nanocomposites due to its substantial reinforcement capabilities and abundant functional groups such as epoxide, carboxyl, and hydroxyl. These functional groups contribute to improved protein adsorption from the extracellular matrix (ECM) and facilitate stem cell attachment, growth, and differentiation. In a recent study by Loyo C. et al., gelatin-coated PCL/GO (1% and 3%) scaffolds were analyzed for their biocompatibility, proliferation, mechanical strength, and hydrophilicity [63]. The adsorption of gelatin (Gt) increased significantly when incorporating graphene oxide (GO) into PCL without the need for fiber surface treatment. The combination of PCL with GO proved to be an effective method for enhancing the bioactivity of PCL, as evidenced by the formation of hydroxyapatite crystals on the coated composites following immersion in simulated body fluid (SBF). Furthermore, biological analysis using human gingival fibroblast stem cells (hGSMCs) indicated that both uncoated and coated PCL and PCL/GO samples supported cell adhesion, with the Gt coating notably enhancing cell proliferation. These findings present a novel approach for fabricating Gt-coated scaffolds by leveraging the advantageous properties of GO as a reinforcing filler. In another innovative study by Niknam Z., durable biocomposite scaffolds were created through the electrospinning technique for the purpose of bone tissue engineering. This involved combining PCL and graphene oxide (GO) with magnesium oxide (MgO) nanoparticles [136].

The periosteum is a resilient connective tissue membrane that envelops bone tissue, serving a crucial function in bone development and healing by supplying progenitor cells, nutrients, and blood flow necessary for fracture recovery. An optimal periosteum exhibits favorable biocompatibility to enhance the attachment and growth of mesenchymal cells, along with robust vascularization capabilities to support the metabolic requirements of cells and bone tissue. The absence of the periosteum and the excessive presence of reactive oxygen species (ROS) at the fracture site contribute to osteoblast aging, leading to reduced proliferation and differentiation, thereby impacting the process of fracture healing. To address this issue, the β-TCP/MnO_2_/PCL artificial periosteum was fabricated via electrospinning, featuring a nanofiber network and a porous structure with loose characteristics [137]. This material possesses the capability to catalyze the decomposition of hydrogen peroxide (H_2_O_2_). The design closely resembles the extracellular matrix structure, thereby enhancing the transportation of blood and essential nutrients. The artificial periosteum developed in this research exhibits enhanced biocompatibility and cell adhesion. When fractures occur and reactive oxygen species (ROS) levels rise, 0.05% to 0.1% by weight of MnO_2_ nanoparticles act as a catalyst to break down H_2_O_2_ and release O_2_, thereby mitigating the impact on osteoblast seed cells such as BMSCs. This process facilitates osteogenic differentiation and fracture healing. Additionally, the findings indicate that an optimal quantity of MnO_2_ nanoparticles can enhance the hydrophilicity of the artificial periosteum and improve cell adhesion. These outcomes suggest that the functionally modified β-TCP/MnO_2_/PCL artificial periosteum represents an excellent material for artificial periosteum applications.

In another study, a tri-layer scaffold was prepared using electrospinning, considering the architecture and characteristics of the natural periosteum [138]. The 100-µm-thick first layer was composed of PCL nanofibers, the 100-µm-thick second layer was composed of PCL/gelatine/Mg-doped ZnO nanofibrous structure, and nanofibrous gelatine/bioglass/COD liver oil made up the 20-µm-thick third layer. The first and second layers of this tissue-engineered periosteum (TEP) were designed to function as vascular layers, while the third layer was designed to fulfill the osteogenic purpose. The average fiber diameters for layers 1, 2, and 3 were 0.475 μm, 0.1 µm, and 0.355 µm, respectively. The incorporation of nanoparticles (doped ZnO) into the angiogenic layer of TEP promotes vascular growth, while COD liver oil in the osteogenic layer supports bone growth. These materials enhance various properties of TEP, including swelling and degradation rates, as well as its morphology and mechanical strength. By integrating these nanoparticles and essential oils into the layer-by-layer structure of TEP, a unique composition is created, paving the way for numerous advancements in periosteum tissue engineering.

In an innovative study by Liu W. et al., the electrospinning technique was used to create an artificial periosteum made from PCL doped with tantalum (Ta) and zinc oxide (ZnO) nanoparticles, aiming to improve its antibacterial, osteogenic, and angiogenic characteristics [139]. In vitro cell studies have shown that the PCL/Ta/ZnO artificial periosteum has outstanding biocompatibility, effectively promoting the osteogenic differentiation of BMSCs and the angiogenic differentiation of endothelial progenitor cells (EPCs). Antibacterial tests revealed its strong bactericidal effects against both *S. aureus* and *E. coli.* In vivo models of subcutaneous infection and critical-sized skull bone defects confirmed its antibacterial properties, ability to promote bone formation, and angiogenic potential. The PCL/Ta/ZnO artificial periosteum is highly effective in controlling infections and positively modulating the immune response, leading to rapid vascularized bone repair.

Polymeric scaffolds play a crucial role in contemporary tissue engineering due to their wide range of options, versatility, and ease of processing. Notably, the physical characteristics of these scaffolds, such as porosity, mechanical strength, and biocompatibility, can be adjusted to create smart, stimuli-responsive materials. In this context, piezoelectric materials can be utilized to promote bone regeneration by transforming mechanical forces into electrical signals. In this context, fibers composed of different blend ratios of polyvinylidene fluoride (PVDF) and PCL (70:30, 50:50, and 30:70) were prepared, studied, and optimized to enhance bone regeneration [112]. Uniform fibers featuring β-phase PVDF were achieved through simultaneous stretching and high voltage during the electrospinning process. PCL exhibited minimal piezoelectric activity; however, the 70PVDF-30PCL blend produced a piezoelectric charge output of approximately 7.5 pC/N. Additionally, combining PVDF with PCL enhanced wettability compared to pure PVDF, leading to improved biodegradability in PBS medium. Tensile test results indicated that both the material composition and structure influence the scaffolds’ mechanical properties. Although adding PVDF to PCL enhances its mechanical performance, the degree of improvement is limited when there is a significantly weak interfacial region, as seen in the 50PVDF-50PCL scaffold. In vitro studies demonstrated that the 70PVDF-30PCL composite scaffold is biocompatible, and its piezoelectric properties facilitated the differentiation of stem cells into osteoblasts. Furthermore, in vitro bioactivity assessments showed apatite formation on the scaffolds, indicating their potential for bone regeneration applications.

**Figure 13:**
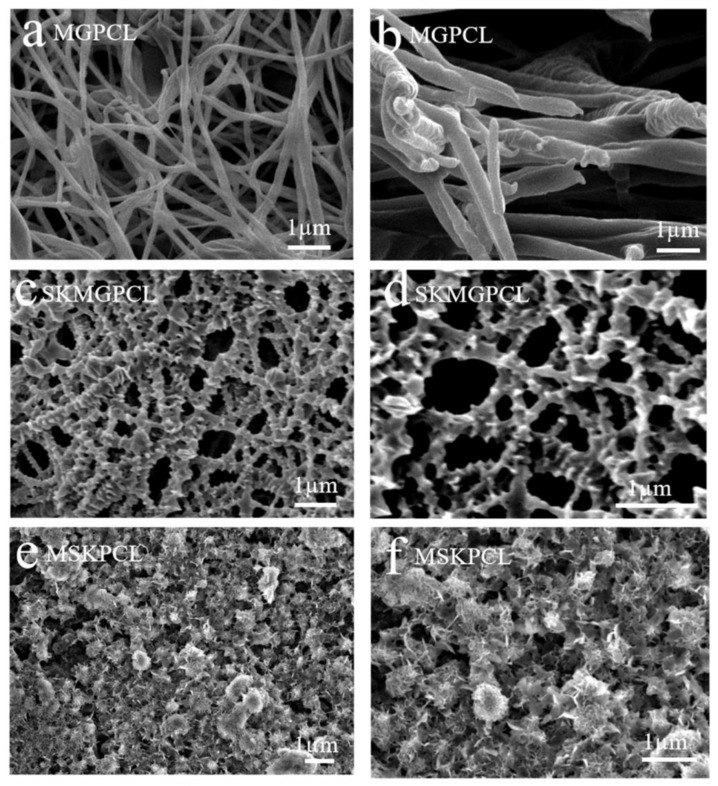
SEM micrographs of different layers of multilayered electrospun fibrous structures. The surface morphology and section view of MGPCL (**a**,**b**), The microstructure of SKMGPCL (**c**,**d**) and MSKPCL (**e**,**f**) [140].

Osteochondral injuries resulting from trauma, tumors, or infections can cause knee joint pain, significantly impacting daily life and ultimately leading to osteoarthritis. Osteochondral tissue is made up of cartilage, calcified cartilage, and subchondral bone. Cartilage itself is classified into three layers: the superficial layer, the intermediate transition layer, and the deep radiative layer. Cartilage injuries are typically categorized into three types based on their depth: partial thickness defects, full thickness defects, and osteochondral defects. Osteochondral defects involve damage to both the cartilage and the underlying subchondral bone. Repairing osteochondral defects is a significant challenge in orthopedics. Hu Y. reported in a recent study that the multilayer fibrous membrane made from PCL for repairing osteochondral defects was biomimetically created using self-induced crystallization, biomimetic mineralization, and layer-by-layer electrospinning methods [140]. The multilayer functional bionic fibrous membrane was made up of a cartilage repair layer, an intermediate transition repair layer, and a subchondral bone repair layer. Glucosamine hydrochloride (GAH) encapsulated in a core-shell structured PCL fibrous membrane (MGPCL) is effective for cartilage repair. A shish-kebab (SK)-structured PCL fibrous membrane with a calcium phosphate coating (MSKPCL) was developed for subchondral bone repair. The SK-structured MGPCL fibrous membrane (SKMGPCL) served as the intermediate transition repair layer. Figure 13 shows the morphology of the prepared layers. The fibrous membrane MGPCL/SKMGPCL/MSKPCL replicates the intricate gradient structure of osteochondral tissue while meeting the mechanical and biological requirements of cartilage and subchondral bone. With a tensile modulus of 34.24 ± 2.39 MPa, it outperforms MGPCL and SKMGPCL fibrous membranes, attributed to tie formation between nanofibers and uniform distribution of calcium phosphate on the nanofiber surface. The SKMGPCL fibrous membrane in the middle transition layer prevents cell migration, separates different cell growth microenvironments, and allows the passage of nutrients and metabolites while preventing slippage. In vitro culture results demonstrate good biological activity and osteogenic ability of the MGPCL/SKMGPCL/MSKPCL fibrous membrane, making it a promising material for osteochondral integrated repair.

Finger joint destruction, particularly in the proximal interphalangeal joint (PIP), can occur due to inflammation, degeneration, or trauma. The standard approach to treatment usually includes managing pain and using nonsteroidal anti-inflammatory drugs (NSAIDs). An optimal prosthetic joint designed for the reconstruction of small joints should meet several key criteria. Firstly, it must possess a combination of strength and flexibility to enable successful implantation and fixation within the joint. Additionally, the artificial joint should exhibit favorable fatigue properties to effectively respond to the mechanical demands associated with finger motion. Furthermore, it should be capable of providing a sustained release of antimicrobial agents and analgesics to prevent infection and alleviate pain. Moreover, the prosthetic joint should be able to deliver appropriate concentrations of growth factors to the targeted areas, thereby promoting the regeneration of bone and connective tissues. It is also essential for the artificial joint to be degradable once it has fulfilled its purpose and to be biocompatible to prevent any tissue irritation during the breakdown process. The reconstruction of small joints continues to present difficulties and can result in complications related to prostheses, primarily attributed to the less-than-ideal performance of silicone materials and negative reactions from the host’s body. To cope with the above requirements, novel hybrid 3D-printed PCL artificial joints were designed and developed [141].

To mimic the complex native tissue, scaffolds with similar architecture and physicochemical properties require the integration of materials science and advanced fabrication techniques. While bone tissue engineering utilizes electrospinning and 3D printing, it is challenging to create 3D structures with electrospun nanofibrous scaffolds due to their dense frameworks, and 3D-printed scaffolds with microscale pores and smooth surfaces struggle to support cell attachment. An innovative approach to address these limitations is to create hybrid scaffolds by integrating fabrication techniques, as demonstrated in a recent study through the fabrication and characterization of hybrid scaffolds by embedding PCL or layered double hydroxides (LDH)/PCL electrospun nanofiber mats into PCL 3D-printed grids [89]. By incorporating LDH into the PCL matrix, modified nanofibrous mats were created with changed physicochemical properties and performance in vitro. The analysis showed that LDH decreased fiber diameter and increased surface roughness of the PCL nanofiber mats while maintaining the accurate dimensions and morphology of the hybrid scaffolds. The LDH/PCL nanofiber mats with hybrid scaffolds demonstrated higher Young’s modulus in compressive tests under both wet and dry conditions, indicating improved mechanical properties. In vitro studies revealed that LDH played a role in enhancing bioactivity and speeding up scaffold degradation, resulting in increased cell adhesion, ALP activity, and calcium deposition in MG-63 cells. The microstructural and biocompatibility characteristics of the LDH/PCL nanofiber mats with hybrid scaffolds suggest potential applications in bone tissue engineering by creating a favorable environment for cell attachment, biomineralization, and promoting osteoconductive properties.

## 5. Conclusions

Researchers have widely employed the fabrication of nanofibrous electrospun PCL-based scaffolds for tissue engineering. The electrospinning technique lends itself to fabricating nanofibers of tunable diameter, orientation, fiber-to-fiber distance, and composition. Electrospun PCL-based scaffolds are commonly blend-electrospun with biomaterials, such as essential oils and natural products, biomolecules, synthetic polymers, drugs, and nanoparticles, to enhance their ability to support tissue growth and repair.

Techniques for the enhancement of PCL-based scaffold properties include multi-scaffold layering, surface functionalization, and coating or hydrogel immobilization. Multi-scaffold layering is relatively simple, as it involves electrospinning one layer on top of another. Surface modification techniques can be used to increase scaffold porosity or to functionalize the scaffold surface with functional groups or biomolecules. Surface coatings and hydrogels can be used to improve the mechanical strength of porous scaffolds.

### 5.1. Challenges and Limitations

Although polycaprolactone (PCL) holds great significance in the biomedical field, it comes with a few limitations. The manufacturing process of PCL is characterized by its complexity and high costs, which have constrained its broader commercialization. A challenge with the electrospinning system is the systematic optimization that is necessary to achieve the desired scaffold nanofiber composition. Furthermore, the hydrophobic nature of PCL results in suboptimal adhesion to cellular structures. Additionally, the solvents utilized in the processing of PCL are recognized for their toxicity, posing potential risks to human health. Another limitation of PCL is its relatively low melting point, which restricts its application in high-temperature environments. Therefore, the integration and processing of polycaprolactone (PCL) into biocomposites assist in enhancing the material characteristics, thereby expanding the potential applications of these superior properties.

### 5.2. Future Work

The development of novel green PCL nanofibers utilizing environmentally friendly solvents and natural biological materials represents a promising avenue for future research aimed at enhancing their applicability in tissue engineering. The growth of publications also presents an opportunity for the incorporation of machine learning to predict nanofiber fabrication conditions. Future iterations of electrospun PCL-based scaffolds may incorporate advanced functionalities, such as electrical conductivity, piezoelectric properties, and responsiveness to various stimuli. Additionally, the investigation of PCL-based Janus fibers presents significant potential for advancements in this domain. Moreover, the application of 3D printing and triaxial electrospinning techniques remains underexplored in the context of tissue engineering. A thorough study of these methodologies could yield substantial improvements in the therapeutic efficacy of PCL scaffolds.

## Figures and Tables

**Table 1:** Overview of benefits and disadvantages of biopolymers and synthetic polymers commonly electrospun with PCL.

Biomaterial	Type	Benefits	Disadvantages	Reference
Gelatin	Biopolymer	Promotes cell attachment and proliferation, supports wound healing, naturally degrades	Globular protein structure causes low spinnability, degrades rapidly, lacks mechanical strength, batch-to-batch variability	[7,8]
Chitosan	Biopolymer	Low risk of immunogenicity, antimicrobial properties reduce risk of infection, supports cell adhesion and proliferation	Low spinnability, lacks mechanical strength, low solubility, batch-to-batch variability, degrades rapidly	[39,40,41]
Collagen	Biopolymer	Provides structural support, naturally degrades, promotes cell adhesion and proliferation	Low spinnability, high-cost, batch-to-batch variability, lacks mechanical strength	[42,43,44]
Silk fibroin	Biopolymer	Promotes cell attachment and proliferation, naturally degrades, mechanically strong	Low spinnability, batch-to-batch variability, high cost	[25,45,46]
Alginate	Biopolymer	Can form hydrogels, naturally degrades, low risk of immunogenicity, low cost	Batch-to-batch variability, limited cell adhesion, may contain impurities	[36,47]
PLA	Synthetic polymer	Low risk of immunogenicity, provides mechanical strength, renewable and biodegradable resource	Temperature sensitive, can be brittle	[26]
PVA	Synthetic polymer	Capacity for high water absorption, easily chemically modified through hydroxyl groups	Limited cell adhesion, hydrophilicity and swelling impact mechanical stability	[21,48]
PEG	Synthetic polymer	Generally biocompatible, water-soluble	Risk of immunogenicity in some individuals, lacks mechanical strength, not biodegradable	[49,50]
PU	Synthetic polymer	Good mechanical strength and flexibility, generally biocompatible	Risk of immunogenicity, byproducts may be inflammatory or toxic	[51]
PVP	Synthetic polymer	Low risk of immunogenicity, water-soluble	Readily absorbs moisture, not biodegradable, lacks mechanical strength	[52,53,54]

**Table 2:** Overview of impacts of scaffold enhancement and post-electrospinning techniques on scaffold properties.

Technique	Impact On PCL-Based Scaffold Properties	Reference
Scaffold multi-layering	Emulate properties of organized multi-level tissue layers, enhance bioactivity or mechanical strength	[45,79]
Acetone treatment	Increase scaffold porosity, improve cell adhesion and infiltration	[80]
Surfactant treatment	Promotes water absorption, cell infiltration into scaffold	[81]
Cross-linked hydrogels	Improved mechanical strength, reduce pore size and improve scaffold hydrophilicity	[36]
Aminolysis treatment	Reduce water contact angle, reduce tensile strength, increased strain, improve bioacticity	[82]
Hydrolysis treatment	Increase fiber diameter, reduce water contact angle, reduce tensile strength and strain, improve bioactivity	[82]
Plasma polymerization	Surface functionalization of biomolecules, reduce water contact angle, and improve bioactivity	[75,83]
Polydopamine polymerization	Increase inter and intramolecular interactions to support bioactivity	[84]
Dip-coating	Modify surface wettability and decorate with biomolecules, reduce water contact angle, modify drug-release profile	[84,85]
